# The critical role of ferroptosis in thyroid cancer development and potential therapeutic implications

**DOI:** 10.3389/fonc.2026.1767384

**Published:** 2026-02-25

**Authors:** Yinghao Li, Tao Qian, Zhongyu Han, Chuchu Wang, Meiqi Zhang, Chi Huang, Qingqing Gu, Shuangyan Zhang, Yumeng Lin, Jianhua Wang, Shouqiang Chen

**Affiliations:** 1 Second School of Clinical Medicine, Shandong University of Traditional Chinese Medicine, Jinan, China; 2 Department of Thyroid and Breast Surgery, Affiliated Hospital of Intergrated Traditional Chinese and Western Medicine, Nanjing University of Chinese Medicine, Nanjing, China; 3 Institute of Nephrology, Zhongda Hospital, Southeast University, Nanjing, China; 4 Graduate School, Tianjin University of Traditional Chinese Medicine, Tianjin, China; 5 Center of Gerontology and Geriatrics, West China Hospital, Sichuan University, Chengdu, China; 6 Nanjing Tongren Hospital, School of Medicine, Southeast University, Nanjing, China; 7 School of Medicine, Shandong University of Traditional Chinese Medicine, Jinan, China

**Keywords:** ferroptosis, immune regulation, therapeutic strategies, thyroid cancer, TME

## Abstract

Thyroid cancer is the most common malignant tumour in the endocrine system, and the global diagnosis rate continues to show a steady upward trend. Although significant progress has been made in surgery, radioactive iodine therapy and Thyroid-Stimulating Hormone (TSH) inhibition therapy, patients still often face the dilemma of tumour recurrence or gradual reduction in efficacy, which makes long-term disease management still challenging. Ferroptosis, as an iron-dependent method of programmed death of cells, has become the core focus of current research. This distinct lethal mechanism is driven by iron-dependent lipid peroxidation. In thyroid cancer research, ferroptosis shows important research value and potential therapeutic significance. This article sorts out the role of ferroptosis in the development and immune regulation of thyroid cancer, explores the mutual influence between it and the environment around the tumour, and focusses on analysing its possible application value in disease diagnosis, prognosis and new treatment development. In general, ferroptosis provides a new research direction for understanding thyroid cancer, and may also open up a new path for clinical treatment. However, at present, most of the relevant evidence comes from basic experiments and animal models. It is still necessary to further study its specific mechanism of action in human body and verify the possibility of its clinical application.

## 1 Introduction

Thyroid carcinoma represents the leading malignancy within the endocrine category. In recent decades, the statistics indicate a notable increase in the number of people affected by this disease (1, 2). Based on the source and cell differentiation level, thyroid cancer is generally categorized into four main histological types: papillary (PTC), follicular(FTC), medullary(MTC), and anaplastic carcinoma (ATC) (3, 4). PTC is the most common type of thyroid cancer. The treatment effect of the vast majority of patients is very good, and the long-term survival rate is very high. 80% to 95% of them can live for more than 10 years. But ATC is the opposite. This kind of cancer is extremely dangerous. It grows and spreads very quickly, so the survival of patients is very bad. It is the most dangerous type of thyroid cancer (5, 6). The exact aetiology of thyroid cancer is not fully clear, but more and more studies have pointed out that radiation exposure, excessive levels of thyroid-stimulating hormone and specific genetic mutations are the main risk factors (1).

Three main factors influence the onset and progression of thyroid cancer: thyroid hormone levels, iodine intake, and genetic susceptibility (7, 8). These factors jointly change the microenvironment around the tumour by affecting the proliferation, differentiation and metabolic activities of cells, thus promoting the development of TC (9–11). At present, the treatment of thyroid cancer mainly adopts a comprehensive treatment plan, which integrates surgical resection, postoperative auxiliary radioiodine-131 ablation treatment and continuous drug TSH inhibition treatment (12). While therapeutic outcomes are generally positive, approximately 20% of cases involve disease relapse or refractory conditions, leading to shortened survival and poorer patient well-being (13, 14). In addition, due to the lack of reliable prognostic biomarkers, it is difficult for doctors to accurately assess the risk level of patients and formulate individualised treatment plans, which makes clinical decision-making particularly difficult.

Ferroptosis is a unique method of programmed cell death, and its occurrence depends on the participation of iron ions (15). Simply put, too much divalent iron ions are accumulated in the cell, and at the same time, the fat molecules in the cell are “over-oxidised”, which eventually leads to cell collapse (16). Research indicates that, besides its involvement in neurological conditions like Alzheimer’s and certain infectious diseases, ferroptosis is implicated in the pathological advancement of diverse malignancies, including breast, lung, hepatic, ovarian, and thyroid neoplasms (17, 18). In thyroid cancer, if the iron and reactive oxygen in cells are increased by drug or genetic methods, or a protective enzyme called Glutathione peroxidase 4 (GPX4) is “turned off”, it can successfully cause ferroptosis in cancer cells, thus effectively inhibiting their growth and spread (19). Conversely, if ferroptosis is prevented in the experiment, the tumour will grow faster, which shows that ferroptosis itself is a natural way for the body to fight tumours (19, 20). Accordingly, regulating ferroptosis with precision could introduce a new way to treat thyroid cancer. Ferroptosis not only participates in the occurrence and development of tumours, but also has the potential to become biomarkers for clinical diagnosis and patient prognosis (21, 22).

The rapid development of tumour biology has gradually clarified the unique morphological characteristics, biological functions and regulatory mechanisms of ferroptosis. As an iron-dependent mechanism of programmed cell death, ferroptosis is involved in numerous elements of cancer progression and treatment. This article will systematically comb out the role of ferroptosis in thyroid cancer, focussing on its key molecular regulatory factors and pathways, interaction with the tumour immune microenvironment, and the potential of related biomarkers in prognosis assessment and treatment. In addition, we will also introduce the latest progress made in the strategy for ferroptosis, including pharmaceutical preparations, natural compounds and combination therapies, and point out the main challenges and future directions for its clinical transformation.

## 2 Pathogenesis of thyroid cancer

More and more evidence shows that the incidence of thyroid cancer continues to rise worldwide, but its specific pathogenesis is not fully clear. Current evidence suggests that the disease follows a multifactorial etiology, driving both its onset and progression.

### 2.1 Radiation

Thyroid tissue is highly sensitive to radiation in the early stage of development, so ionising radiation exposure is a clear pathogenic factor of thyroid cancer, especially prominent in children (23). Initial reports linking pediatric exposure to thyroid carcinoma appeared in the 1950s: it was reported that 10 of the 28 thyroid cancer patients aged 18 and under had received X-ray treatment for thymus hyperplasia within 4–16 weeks after birth (24). This relationship was corroborated following the Chernobyl meltdown in 1986, when widespread radioactive contamination drove a surge in thyroid carcinoma diagnoses among children and adolescents residing in the vicinity (25). Additionally, diagnostic procedures such as dental X-rays and CT scans, as well as radiotherapy to the head, neck, or chest, are also considered to increase the risk of thyroid cancer (26, 27).

Ionising radiation triggers oxidative stress in biological systems through direct and indirect means, and affects local and systemic processes through a variety of mechanisms, including accelerating ageing, causing genetic instability and mutation, causing cell membrane damage and cell death, changing enzyme function and cell metabolism, damaging mitochondrial function and initiating the cancer process (28, 29). Further research shows that ionising radiation may trigger RET/PTC gene rearranment (30). In thyroid papillary carcinoma, the RET proto-ocarcinoma gene can produce a variety of chimeric oncogenes, which are collectively known as RET/PTC. RET/PTC rearraising is a key molecular event, and its frequency has been confirmed to increase with the increase of radiation dose among atomic bomb survivors (31). Therefore, reducing ionising radiation exposure, especially among children and adolescents, may play a key role in the prevention of thyroid cancer.

### 2.2 Gene mutations

**Figure 1:**
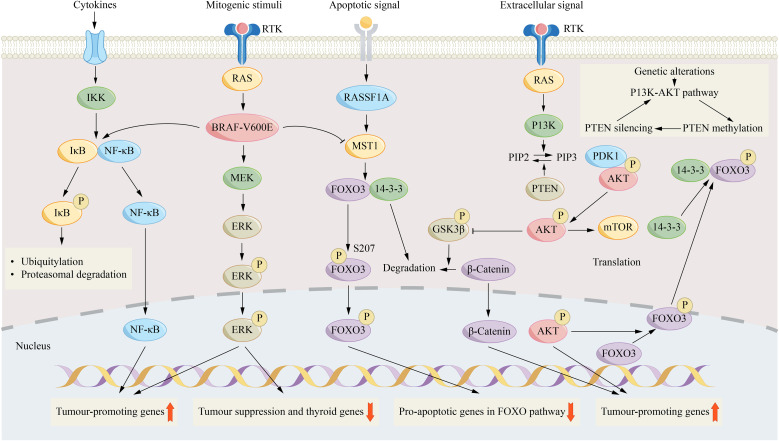
Key signaling pathways in thyroid cancer. This schematic illustrates three core pathways in thyroid tumorigenesis. The central MAPK pathway, initiated by mitogenic stimuli via receptor tyrosine kinase (RTK)/RAS, is hyperactivated by the BRAF-V600E oncoprotein, leading to ERK phosphorylation and nuclear translocation to modulate gene expression. BRAF-V600E independently activates the nuclear factor-kB (NF-κB) pathway (left) by promoting IκB phosphorylation and degradation, enabling NF-κB to transcribe pro-tumor genes. Simultaneously, BRAF-V600E suppresses the pro-apoptotic RASSF1A-mammalianSTE20-like protein kinase 1 (MST1)- forkhead box 03 (F0X03) pathway by inhibiting MST1, thereby blocking FOXO3-mediated apoptosis. On the right, PI3K-AKT signaling, activated by RTK/RAS, promotes tumorigenesis through multiple mechanisms: phospho-AKT activates mTOR, inhibits GSK3β to stabilize β-catenin, and directly phosphorylates nuclear FOXO3, leading to its cytoplasmic sequestration and inactivation. The pathway is negatively regulated by PTEN, and an inset highlights a self-enhancing loop whereby pathway activation promotes PTEN silencing via methylation, sustaining constitutive signaling. DAPK1, death-associated protein kinase 1; HF1A, hypoxia-inducible factor 1a; MMP, matrix metalloproteinase, NIS, sodium-iodide symporter, TGFB1, transforming growth factor B1; TIMP3, tissue inhibitor of metalloproteinases 3; TPO, thyroid peroxidase; TSHR, thyroid-stimulating.

Accumulating evidence identifies BRAF activating mutations and RET/PTC rearrangements as central drivers in the pathogenesis and progression of papillary thyroid carcinoma (**Figure 1**). Thyroid cell division and maturation are governed by BRAF, a specific serine/threonine kinase. The mutation of this gene will drive the occurrence and development of tumours (32, 33). Among them, the T1799A point mutation of the BRAF gene will produce a BRAF-V600E variant, causing the kinase to be in a state of continuous activation (34, 35). Approximately 45% of papillary thyroid carcinoma cases harbor the BRAF-V600E mutation, and the mutation is closely related to adverse clinical pathological characteristics, including invasive histological types, higher risk of recurrence and reduced reactivity to radioactive iodine treatment (36, 37). RET/PTC rearrangements, among the earliest genetic events in thyroid tumorigenesis, are exclusively identified in PTC and occur in 20–40% of sporadic adult tumors (30, 38).

### 2.3 Abnormal hormone levels

TSH is a well-known stimulator of thyroid cells. Excess TSH continuously stimulates follicular cells, leading to their hypertrophy and proliferation, and ultimately undergoing malignant transformation through stages of nodules, focal proliferation, and adenomas (39). Multiple studies have recognized serum TSH as an independent marker for diagnosing thyroid malignancies, with higher TSH concentrations correlating with increased cancer risk and greater tumor aggressiveness in well-differentiated thyroid cancer (40, 41). Despite remaining within standard limits, this factor is linked to increased cancer susceptibility and accelerated tumor growth (42, 43).

There is a significant gender difference in thyroid cancer: the rate of women suffering from the disease is 2.9 times that of men (44). The carcinogenic effect of oestrogen is mainly realised by its binding to the receptors ER-α and ER-β, and these complexes then interact with oestrogen-reactive elements, thus activating multiple cell survival pathways (45). Therefore, the endoplasmic reticulum has become a key cell and transcription regulation hub in the proliferation mechanism of endocrine-related malignant tumours. Studies show that oestrogen can promote the proliferation of human thyroid cancer cells by activating the filament activated protein kinase (MAPK) pathway (46). Estrogen may also stimulate transfer phenotypes, including adhesion and migration, by inhibiting the components of the calcium mucin-cyclic protein system (47).

### 2.4 Iodine intake

Iodine intake is closely related to the frequency of detection of thyroid cancer. Data shows that in iodine-deficient areas, the proportion of invasive ATC and FTC is higher (48). In the normal physiological state, the proliferation rate of thyroid follicular cells is slow. However, when iodine intake is insufficient, elevated serum thyroid-stimulating hormone (TSH) will strongly stimulate its proliferation, increasing its growth rate by 5 to 30 times, resulting in significant follicular cell proliferation and glandular enlargement (49). When thyroid cells are in a state of rapid proliferation, their sensitivity to mutagenic factors such as radiation, chemical carcinogens and oxidative stress may be significantly enhanced, making it easier to accumulate additional genetic mutations.

On the contrary, it is also important to note that excessive intake of iodine can be harmful. Excessive iodine consumption exacerbates the risk of dysfunction, particularly in subjects with a history of thyroid pathology or antecedent iodine deprivation (50). Thyroid dysfunction associated with iodine excess is most often clinically insignificant and reversible, whereas iodine-induced hyperthyroidism may, in susceptible individuals, reach a life-threatening severity. Evidence has indicated that excessive iodine consumption may be associated with thyroid carcinogenesis, potentially mediated by enhanced oxidative damage to DNA (51). Therefore, more long-term large-scale case-control study data is needed to accurately estimate iodine intake in order to evaluate the potential relationship with iodine deficiency and excessive iodine intake.

### 2.5 Lipid metabolism

Lipid metabolism in tumors is primarily characterized by significant reprogramming of fatty acid (FA) metabolism. Disruption of the balance between FA synthesis and degradation can profoundly affect key hallmarks of tumor biology, including, but not limited to, cancer cell migration, proliferation, apoptosis, and local invasion (52). Dysregulation of fatty acid metabolism contributes to thyroid cancer development. Upregulation of stearoyl-CoA desaturase 1 (SCD1) is evident in both ATC and well-differentiated subtypes, serving a critical function in maintaining ATC cell viability and growth (53). Acetyl-CoA carboxylases (ACCs) are rate-limiting enzymes in the cytoplasm that regulate fatty acid metabolism. Research indicates that circPCNXL2 is overexpressed in PTC tissues and cells, promoting cell proliferation by inhibiting ACC1 phosphorylation and enhancing fatty acid synthesis (54). Therefore, given that the high progression of thyroid cancer is closely related to lipid metabolism disorders, intervention in these metabolic pathways has become a highly potential treatment direction.

### 2.6 Immune system anomalies

In the tumour microenvironment (TME) of thyroid cancer, macrophages, as the main interstitial component, have a double regulating effect on the course of the disease: they can both inhibit and promote the development of cancer (55, 56). T In the TME of thyroid cancer, tumour-assiated macrophages (TAMs) usually differentiate into two phenotypes with very different functions: M1 and M2. In the early stage of cancer, M1 macrophages play the role of clearing cancer cells and activating immune responses; while M2 tends to promote tumour growth and spread. Current research generally believes that the functional imbalance of the TME is an important driving force for the progression and metastasis of thyroid cancer (57). Although the body’s immune system can effectively remove early tumour cells, the TME will eventually help cancer cells escape immune attacks by inhibiting the killing ability of immune-effecting cells (58–60). The infiltration density of M2 macrophages is significantly positively correlated with the malignant progression of thyroid cancer, which makes the cell group a highly potential key treatment target (61, 62).

## 3 Mechanism of ferroptosis

Ferroptosis represents a unique modality of regulated cell mortality, distinguished specifically by its reliance on iron-catalyzed lipid oxidation. Its specific morphological and mechanistic traits clearly separate it from other established pathways, such as apoptosis, necroptosis, and pyroptosis (16, 63). Morphologically, cells with ferroptosis will show mitochondrial contraction, mitochondrial membrane density increase and ridge structure disappearance. Mechanistically, the majority of regulated cell death modalities depend on dedicated signaling cascades that mobilize executioner molecules or pore-forming agents, such as caspases in apoptosis, MLKL in necroptosis, and gasdermins in pyroptosis (64, 65). In contrast, the occurrence of ferroptosis does not depend on such pore formation or execution proteins. It is mainly due to cell metabolism disorders, which causes phospholipids containing polyunsaturated fatty acids to oxidise and accumulate, thus triggering cell death (66).

**Figure 2:**
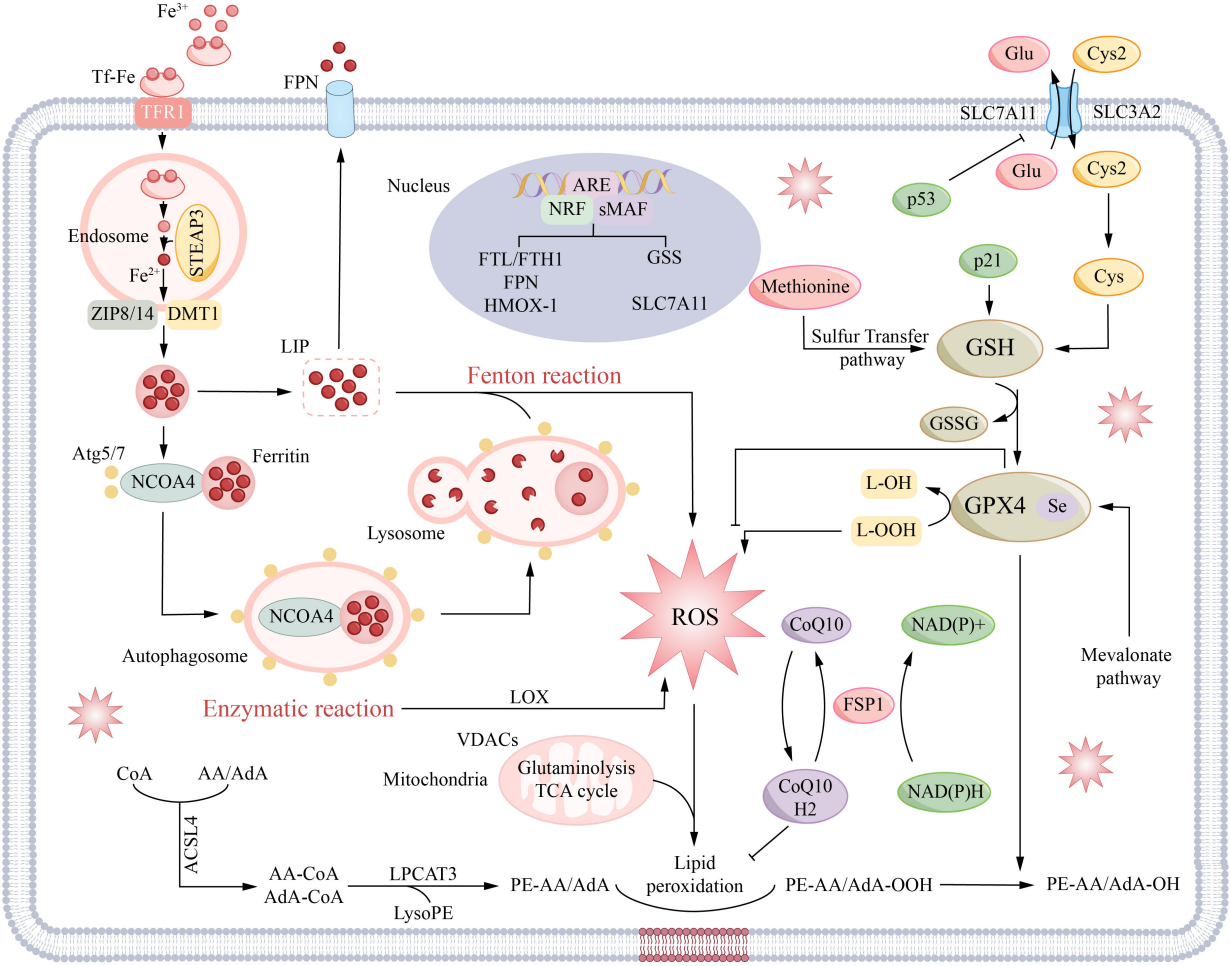
The core molecular mechanisms and signal regulation of ferroptosis. The core metabolic processes of ferroptosis can be roughly summarized into the following three aspects: iron metabolism, GSH/GPX4 signaling pathway and lipid peroxidation. In addition, mitochondria play an important role in ferroptosis induced by cystine deficiency. NRF2 can promote the expression of factors involved in iron metabolism (such as FTL/FTH1) and iron transporter (FPN) and regulate the synthesis of glutathione by up-regulating the expression of glutathione synthetase (GSS) and other genes. Meanwhile, NRF2 also promoted the expression of SLC7A11. In addition, recent studies have shown that FSP1-CoQ10-NAD(P)H pathway acts as an independent parallel system, synergistically with GPX4 and glutathione to inhibit the occurrence of phospholipid peroxidation and ferroptosis.

During ferroptosis, the accumulation of ferrous ions (Fe²^+^) within cells catalyzes the Fenton reaction, generating one of the most reactive oxygen species (ROS)—the hydroxyl radical (67, 68). Escalating concentrations of ROS compromise membrane integrity by targeting polyunsaturated fatty acids, resulting in the accrual of lethal lipid peroxides (69, 70). Lipid peroxides in cells are mainly removed through the synergy of glutathione and GPX4 (15). GPX4 takes glutathione as a cofactor to convert harmful lipid peroxides into harmless liposols, thus effectively neutralising the oxidative pressure in the cell (71). On the contrary, the impaired function of GPX4 will trigger the out-of-control of the lipid peroxidation reaction, which will eventually trigger the process of ferroptosis (69). This section focusses on the specific mechanism of iron, p53, amino acid and lipid metabolism network in the ferroptosis cascade reaction, and takes into account the regulatory role of coenzyme Q, GTP cyclised hydrolase 1 and mitochondrial voltage-dependent anion channel (**Figure 2**) (72, 73).

### 3.1 Iron dynamics in ferroptosis

The iron transport system in the cell precisely regulates the steady-state balance of iron. If this system fails, it may lead to iron overload, which in turn becomes a key factor in iron poisoning (74). Physiologically, iron serves as a fundamental trace element, present primarily in Fe^2+^ or ferric (Fe^3+^) states (75). Following intestinal uptake or the breakdown of erythrocytes, the plasma ferroxidase ceruloplasmin oxidizes Fe^2+^ species into the Fe^3+^ form (76). Following this, iron combines with transferrin to form a complex, which is taken up by cells via transferrin receptor 1 (63). In the endosome, metal reductase STEAP3 reduces iron ions to Fe^2+^, and then transports them to the cytoplasm by divalent metal ion transporter 1 (77). After entering the cytoplasm, iron can enter the dynamically unstable iron pool or be stored in ferritin - ferritin is a polymeric assembly structure composed of light chains and heavy chains (78, 79). Surplus cellular iron is eliminated via Ferroportin, the efflux channel encoded by SLC40A1, which facilitates the translocation and subsequent oxidation of iron ions (80).

The precise absorption, storage and release regulation of iron is the key to maintaining the iron homeosis of cells (81). Once this dynamic balance is broken, it will cause intracellular iron overload. Excess iron ions will catalyse lipid peroxidation through Fenton reaction and Haber-Weiss cascades, thus irreversibly triggering ferroptosis (77, 79). The latest research shows that disorders of iron metabolism can lead to excessive accumulation of iron elements in cells, which has been proven to be a key driver of inducing ferroptosis (82, 83). Elucidating the molecular controls governing iron metabolism is essential for defining the fundamental pathways of ferroptosis (84).

### 3.2 Lipid metabolism and ferroptosis

The characteristic feature of ferroptosis is the fatal accumulation of lipid peroxide. Its toxicity mainly comes from the oxidation of polyunsaturated fatty acids (PUFAs) (85). PUFAs, especially arachidonic acid and prostaglandin E2, are the main targets of lipid peroxidation reactions. The oxidation process can be initiated by specific enzyme catalysis or spontaneous non-enzymatic chemical reactions (86, 87). In the enzyme catalytic pathway, arachidonic acid is first activated into AA-CoA under the action of ACSL4, and then AE-PE is esterified by LPCAT3 and phosphatidylethanolamine. The complex undergoes an oxidation reaction under the catalysis of lipoxygenase or cytochrome P450 oxidase, and finally produces lipid peroxide (88, 89). The Fenton reaction promotes the non-enzymatic lipid oxidation reaction by producing highly active hydroxyl radicals (90). The accumulation of these toxic substances will change the biophysical properties of cell membranes and cause oxidative damage to intracellular biological macromolecules and organelles, eventually causing iron-dependent cell death.

Inhibiting the activity of ACSL4 or LPCAT3 can reduce the accumulation of phospholipid peroxide and make cells resistant to ferroptosis (91). In contrast, monounsaturated fatty acids (MUFAs), such as oleic acid, limit the integration of polyunsaturated chains into membrane lipids through competitive assimilation. This competitive displacement limits the substrate available for oxidation, effectively dampening cellular susceptibility to ferroptosis (92). Dissecting the functional interplay among ACSL4, LPCAT3, LOXs, and POR is fundamental to decoding the etiology of ferroptosis and pinpointing new strategies for intervention.

### 3.3 Amino acid metabolism and ferroptosis

Amino acid metabolism exerts substantial control over ferroptosis, primarily by governing the System Xc^-^–glutathione–GPX4 signaling axis (93). The Xc^-^ system, composed of the SLC7A11 and SLC3A2 subunits, serves as a cysteine/glutamate antiporter. Its primary physiological role is to facilitate the export of intracellular glutamate in exchange for the import of extracellular cystine (16). Following internalization, cystine undergoes reduction to form cysteine, thereby supplying the rate-limiting substrate for glutathione biosynthesis. Subsequently, the sequential catalytic action of glutamate-cysteine ligase and glutathione synthase assembles glutamate, cysteine, and glycine into glutathione, the obligatory cofactor for GPX4 (94). As a key phospholipid peroxidase, GPX4 uses glutathione to reduce toxic phospholipid peroxides into harmless alcoholic substances, thus protecting the cell membrane structure and effectively inhibiting the occurrence of ferroptosis (66).

When using substances such as elastin, sorafenib, sulfasalazine, or high concentration of glutamic acid to block the Xc− function of the system, it will interfere with the uptake of cysteine, reduce the level of glutathione in cells, inhibit GPX4 activity, lead to the accumulation of phospholipid peroxide and cause ferroptosis (94, 95). Similarly, direct inhibition of GPX4 (such as the use of RSL3) will also destroy the redox homeostas and promote the occurrence of fatal lipid peroxidation (96). In addition to this classical pathway, other metabolic pathways also participate in the regulatory network of ferroptosis, including the transsulphur pathway of converting methionine into cysteine, and the regulatory role of glutamine catabolism. These mechanisms together reveal the multi-level regulation of amino acid metabolism to ferroptosis sensitivity in normal cells and malignant cells (97, 98).

### 3.4 Antioxidant system and ferroptosis

Several pathways suppress lipid peroxidation through distinct mechanisms, primarily the ferroptosis inhibitory protein 1(FSP1)–CoQ10, mitochondrial DHODH–CoQ10, and GPX4 systems, and their inactivation directly contributes to the occurrence of ferroptosis. FSP1, located at the plasma membrane, reduces CoQ10 to its active form using NAD(P)H, scavenging lipid peroxyl radicals independently of the classical GSH–GPX4 pathway (99). Evidence suggests that FSP1 functions in tandem with GPX4 to orchestrate the control of ferroptosis within retinal pigment epithelial tissues (100, 101). In mitochondria, besides GPX4, DHODH also reduces CoQ10, preventing ferroptosis (102). Together, GPX4 (cytoplasm and mitochondria), FSP1 (plasma membrane), and DHODH (mitochondria) form three complementary antioxidant defense systems (103).

It is worth noting that the functions of DHODH and GPX4 in mitochondria can compensate for each other, but cytoplasmic GPX4 or plasma membrane FSP1 cannot replace the mitochondrial protective function of DHODH (104, 105). On the contrary, the impaired function of DHODH can promote ferroptosis. Type I interferon, for instance, enhances manganese-induced ferroptosis via enzyme inhibition, whereas DHODH silencing can restrain the expansion of cervical cancer cells and promote ferroptotic cell death.

Beyond these canonical pathways, the cellular antioxidant repertoire includes several alternative defense systems. For example, the GCH1-mediated tetrahydrobiotic (BH4) synthesis pathway can not only directly remove lipid peroxides, but also promote endogenous coenzyme Q10 synthesis through membrane remodelling, thus indirectly inhibiting ferroptosis. Coenzyme Q10 is an endogenous lipid with redox activity. Its side chain is composed of ten isopentane units, which is crucial to its function of maintaining cell homeostasis (100). Moreover, depletion of CoQ10H_2_ directly induces ferroptosis (106). Collectively, these studies demonstrate a close association between antioxidant systems and the regulation of ferroptosis.

### 3.5 P53 and ferroptosis

As one of the most important cancer suppressor genes, p53 regulates many key life activities such as cell cycle blockage, ageing, apoptosis and ferroptosis, thus effectively inhibiting the occurrence and development of malignant tumours (107, 108). Under oxidative stress conditions, p53 will participate in the regulation of cell death, and its effect will converge in ferroptosis and apoptosis pathways. For example, in lung cancer cell experiments, it was found that when the p53 gene is silenced, most of the cells can still survive under reactive oxygen exposure, with a survival rate of more than 90%; on the contrary, when reactive oxygen activates p53, the cell death rate will rise to about 40%, and this death effect can be affected by the ferroptosis inhibitor Fer- 1 Significantly prevent. This shows that the activation of p53 will guide cells towards ferroptosis under certain conditions (109).

P53 regulates ferroptosis through a variety of molecular mechanisms. By suppressing SLC7A11, p53 reduces cystine import through system Xc^-^, weakens GPX4 activity, and promotes ROS accumulation, ultimately inducing ferroptosis (107, 110). Moreover, as described by Ou et al. (2016), p53 can facilitate ferroptotic cell death through the SAT1–ALOX15 axis (111). In this study, the induction of SAT1 did not affect the expression level of GPX4, and the overexpression of SLC7A11 could not save cell death, indicating that this pathway was independent of GPX4 and system XC ^-^. Further experiments showed that SAT1 depended on ALOX15 (an enzyme catalyzing arachidonic acid peroxidation) to trigger ferroptosis, which could be effectively blocked by an ALOX15 inhibitor.

However, some studies also pointed out that p53 may inhibit ferroptosis under certain conditions. Tarangelo et al. (2018) found that stable expression of wild-type p53 decreases cellular susceptibility to ferroptosis and diminishes system Xc^-^ activity (112). Based on studies of CDKN1A (p21), it has been proposed that p53, via the p21 pathway, can restrict ferroptotic cell death in tumors. This highlights the dual role of p53 in ferroptosis: both negative regulation by inducing p21 and positive regulation by inhibiting the system Xc−. The regulatory effect of p53 on ferroptosis is highly situationally dependent, and it is dynamically regulated by the cell microenvironment and the dominant signalling pathway, so its precise mechanism still needs to be continuously explored (113).

### 3.6 Mitochondrial VDACs and ferroptosis

The voltage-dependent anion channel (VDAC), residing in the mitochondrial outer membrane (MOM), mediates both the exchange of ions and metabolites and the generation of ROS (114). Abnormal function of the VDAC channel contributes to the execution of ferroptotic cell death. Blocking VDAC oligomer formation mitigates the accretion of both intracellular iron and reactive oxygen species, thereby conferring cytoprotection against ferroptosis via the curtailment of lipid oxidation (115). Especially in the mouse model, inhibitors such as vbit-12 and fer-1 showed protective effects. In addition, Yagoda et al. (2007) demonstrated that the direct association of erastin with VDAC induces mitochondrial dysfunction and subsequent oxidative burst, thereby initiating the ferroptotic process (116). Specific isoforms, particularly VDAC3, have been implicated in the iron-dependent cell death pathways activated by erastin and RSL5 (117), although the specific mechanism is not completely clear (118).

Beyond the VDAC axis, the regulation of ferroptosis encompasses several secondary mechanisms, including sulphur metabolism, heme oxygenase-1 activity, transferrin routing, and the peroxidative cascade of polyunsaturated fatty acids. Iron ions potentiate the accrual of lipid reactive oxygen species, and this combination of factors collectively drives the onset of cellular demise (119). In essence, VDAC is prerequisite to the convergence of mitochondrial metabolism and oxidative stress; however, its precise role in the ferroptosis pathway still awaits thorough elucidation.

## 4 Ferroptosis and the TME

**Figure 3:**
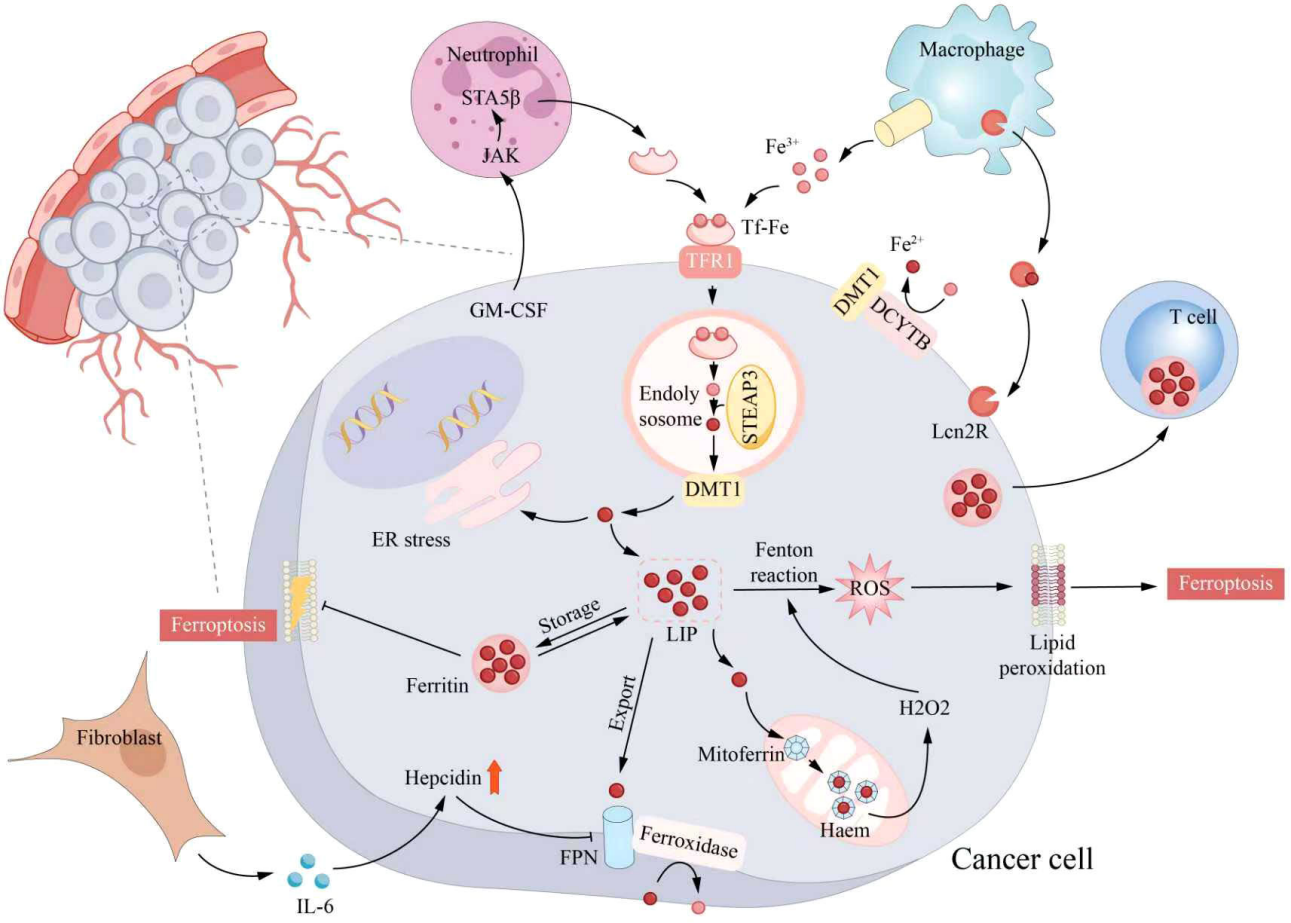
Role of tumor microenvironment (TME) components in ferroptosis regulation. Iron, bound to transferrin (TF), enters the cell via transferrin receptor 1 (TFR1)-mediated endocytosis. In the acidified endolysosomal compartment, iron is reduced by STEAP3 and transported into the cytosol through divalent metal transport-1 (DMT1), contributing to the labile iron pool (LIP). The LIP serves as a dynamic iron reservoir: iron can be stored within ferritin nanocages, utilized in mitochondria for oxidative respiration, or exported extracellularly via ferroportin (FPN) in conjunction with ferroxidase activity. Hepcidin production is increased by IL-6 released from cancer-associated fibroblasts (CAFs), which blocks FPN-mediated iron export and encourages the retention of iron within cells. Increased LIP levels promote Fenton reactions, producing reactive oxygen species (ROS) that lead to lipid peroxidation and initiate ferroptosis. TFR1 and STEAP3 expression is frequently increased in cancer cells. Moreover, M2-like macrophages in the tumor microenvironment can provide additional iron to cancer cells, aiding their increased metabolic needs and vulnerability to ferroptosis. STEAP3, steap3 family member-3; CAFs, cancer-associated fibroblasts; Lcn2, lipocalin 2.

Recently, people have paid more and more attention to the ferroptosis, which is due to the recognition that it is an inherent tumour inhibition process and is hoped to become a targeted way to stimulate anti-tumour immunity. The TME is a heterogeneous whole composed of malignant cells, immune cells, matrix cells and other types of cells, and the mutual influence between these cells drives the behaviour of tumours. Ferroptosis participates in this network in a two-way way: the ferroptosis of cancer cells will produce metabolic and inflammatory signals, thus reshaping the local immune response; and the immune cell group in the TME has different susceptibility to ferroptosis, which can both promote and counteract the execution process of ferroptosis of tumour cells (**Figure 3**).

### 4.1 Regulatory functions of TME components in ferroptosis

Within the TME, diverse cell populations modulate ferroptotic susceptibility, functioning to either suppress or drive the pathway. For instance, CD8+ T cells sensitize tumors to ferroptosis through the secretion of interferon-γ (IFNγ) (120, 121). In terms of mechanism, IFNγ simultaneously downregulates SLC7A11 to limit the intake of cysteine, and upregulates ACSL4 to promote the biosynthesis of polyunsaturated phospholipids rich in ararachidic acid. This process weakens the antioxidant ability and promotes lipid peroxidation at the same time, eventually leading to the death of ferroptotic cell death. Therefore, the simultaneous existence of IFNγ and arachidonic acid in the TME has become a key determinant of ferroptosis in cancer cells (120, 121).

In addition to CD8+ T cells, neutrophils also promote ferroptosis by transferring their particles containing medullary peroxidase to tumour cells (120). In contrast, cancer-related fibroblasts (CAFs), which have a profound impact on both congenital and adaptive immunity, tend to inhibit ferroptosis (122). This function of CAFs can be achieved not only by increasing the expression of long-stranded non-coding RNA DLEU1, but also by releasing miR-522 (123). In gastrointestinal tumors, anoctamin 1 inhibits ferroptosis and elevates TGF-β release, which attracts CAFs and weakens CD8^+^ T cell–driven antitumor immunity, thereby promoting immunotherapy resistance (124). These findings highlight the reciprocal interactions among tumor cells, fibroblasts, and immune cells in dictating ferroptotic outcomes.

Cytokine signalling further increases this complexity. TGF-β1 will activate the signalling pathway that depends on SMAD, thus promoting the ferroptosis of cancer cells; while interleukin-1β (IL-1β) enhances the production of NADPH and stabilises iron-sulphur clusters by acetylation of niacinamide nucleotide transhydrogenase, thus inhibiting ferroptosis (125, 126).

In addition to cytokines, metabolic signals in the TME also play a decisive role. This environment is usually characterised by high accumulation of lactic acid and limited glucose availability (127, 128). Under the condition of glucose deficiency, AMPK will phosphorylate acetyl coenzyme A carboxylase to maintain resistance to ferroptosis (129, 130). At the same time, excessive lactic acid not only inhibits immune function, but also enhances the resistance of tumour cells to ferroptosis by stimulating the synthesis of monounsaturated phospholipids or further acidifying the TME (131, 132). Interestingly, acidosis is not universally inhibits ferroptosis; cancer cells in an acidic environment are particularly vulnerable to ferroptosis induced by n-3 and n-6 polyunsaturated fatty acids (133).

In a word, the regulation of ferroptosis in the tumour is an interaction between cytokines derived from immunity and matrix and the metabolic status of TME changes, depending on the specific situation. Future treatments will need to use the properties that promote ferroptosis while offsetting its resistance mechanism, so as to shift the balance to ferroptosis in favour of cancer cells.

### 4.2 Balancing antitumor immunity via ferroptosis

Macrophages, neutrophils, and natural killer (NK) cells are the principal constituents of the innate immune system, forming the frontline immunological barrier against tumor development (134). In addition to directly killing malignant cells, they also coordinate and enhance adaptive immune responses. However, their function is strictly regulated by the ferroptosis and the biologically active metabolites it produces (135). In the TME, tumour-related macrophages (TAMs) mainly exhibit immunosuppressive M2 phenotypes, which prompts extensive research to focus on reducing the number of M2 type TAMs or reprogramming them into anti-tumour M1 states (136). Recent findings demonstrate that M1 TAMs exhibit enhanced resilience to ferroptosis compared to M2 TAMs, suggesting that susceptibility to this cell death modality is dictated by the macrophage functional state (137).

The differential vulnerability of tumor-associated macrophages (TAMs) to ferroptosis is attributable, in part, to their distinct metabolic profiles and molecular identity. The different susceptibility of M1 and M2 TAMs to ferroptosis can be partly attributed to the differences in their metabolism and molecular properties. These free radicals can effectively remove lipid free radicals produced by the 15-lipooxygenase (15-LOX) pathway, thus protecting M1 type TAMs from ferroptosis (137). In addition, M1 macrophages showed an increase in the level of ferritin heavy chain 1 - which contributes to ferritin assembly and intracellular iron storage - at the same time, the level of iron-output transporter iron transporter is reduced (138). These changes work together to reduce the unstable iron pools in M1 TAMs, thus enhancing their inherent resistance to ferroptosis.

Given their heightened vulnerability, M2 TAMs are more susceptible to ferroptosis than M1 subtypes. This suggests a therapeutic strategy for the selective elimination of immunosuppressive M2 macrophages while sparing the M1 population. Such approaches not only purge the immunosuppressive population but also potentially re-engineer the surviving macrophages toward an anti-tumor M1 phenotype, consequently boosting the therapeutic power of cancer immunotherapy (139). Supporting this concept, many studies have shown that iron nanoparticles, a powerful ferroptosis inducer, can drive the transformation of M2 TAMs into M1-like cells, ultimately enhancing their anti-tumour function (140, 141).

In addition to the method of nanoparticle-mediated, the signalling pathway also has a key impact on ferroptosis and macrophage polarisation. For example, receptor tyrosine kinase TYRO3 has become a key regulatory factor in the TME (142). TYRO3 fosters an immunosuppressive microenvironment through a dual mechanism: counteracting ferroptosis in cancer cells and shifting the M1/M2 macrophage ratio towards the M2 subtype. Conversely, pharmacological blockade of TYRO3 simultaneously compels tumor cells into ferroptosis, shifts macrophage polarization toward the anti-tumor M1 state, and potentiates the therapeutic response to PD-1 checkpoint inhibition (142).

However, emerging evidence indicates that the relationship between tumour-associated macrophages (TAMs) and ferroptosis is more complex and subtle than previously recognised. In some contexts, ferroptosis promotes the accumulation of TAMs and drives their polarisation toward an immunosuppressive M2 phenotype, whereas blocking ferroptosis has been shown to hinder this M2 polarisation. This context-dependent effect has been observed in glioblastoma models, highlighting the dual and situational roles of ferroptosis in regulating TAM behaviour (143, 144).

In short, these studies highlight a complex and situational relationship between ferroptosis and macrophage polarisation, revealing the seemingly contradictory effect of ferroptosis against tumour immunity.

In the TME, medullary inhibitory cells (MDSCs) are the main cell groups that actively inhibit immune response (145). The significant ferroptosis resistance of these cells is mainly due to the inhibitory effect of ASAH2 on the p53–HO-1 signalling axis (146). Targeting ASAH2 by drug or genetic means can trigger ferroptosis in MDSCs, which then enhances the infiltration of CD8+ T cells and strengthens the anti-tumour immune response (146).

Attention has also been paid to the prospective advantages of combining methods that promote ferroptosis with strategies targeting MDSCs. In the mouse model of liver cancer, the deletion of GPX4 increased the infiltration of CD8+ T cells. However, it also increases the expression of PD-L1 on tumour cells and promotes the collection of polymorphic nuclear MDSCs, ultimately limiting the tumour inhibition effect (147). Importantly, the combined use of GPX4 inhibitors and the strategy of inhibiting the recruitment of MDSCs significantly improved the therapeutic effect of anti-PD-1 and prolonged the survival of mice with GPX4 wild tumours (147).

Recent evidence shows that polymorphic nuclear medullary inhibitory cells (PMN-MDSCs) play a role that depends on specific situations and sometimes seems contradictory in the regulation of ferroptosis (148). In the TME, PMN-MDSCs themselves are prone to ferroptosis, partly due to hypoxia leading to the downward regulation of GPX4 expression. Paradoxically, inducing ferroptosis of such cells may enhance their immunosuppressive activity, which is likely to be achieved by releasing phospholipid oxide that can inhibit the reaction of T cells. Therefore, inhibiting ferroptosis rather than inducing ferroptosis may more effectively inhibit tumour growth by reducing the immunosuppressive effect mediated by PMN-MDSCs; while the use of ferroptosis inducers for treatment may inadvertently accelerate the progression of the tumour (148).

It is worth noting that some observations differ from most of the findings obtained in the immunosound mouse model. Although most studies show that ferroptosis, which triggers immunosuppressive cells, can enhance the body’s anti-tumour immune response, and systemic administration of ferroptosis inducers in immune-healthy mice can usually inhibit tumour progression, several significant exceptions have been reported. For example, those neutrophils that infiltrate tumours and exhibit immunosuppressive characteristics similar to PMN-MDSCs show strong resistance to ferroptosis (149). The observed ferroptosis resistance is related to the up-regulation of acetic acid decarboxylase 1 (ACOD1), which can promote the synthesis of lycolic acid and trigger the protection mechanism mediated by NRF2 against ferroptosis. On the contrary, the deletion of ACOD1 will reduce the recruitment of neutrophils, enhance the anti-tumour immune response, and enhance the effect of immunotherapy intervention. The root cause of these differences may be due to differences in experimental design, differences in animal models, or the intrinsic heterogeneity of PMN-MDSCs and tumour-related neutrophils themselves. This highlights the urgent need for in-depth research on how ferroptosis regulates the TME and thus affects the anti-tumour immune response.

NK cells are another vital part of the intrinsic immune system (150). In the TME, the oxidative stress caused by lipid peroxidation is related to NK cell disfunction. Activating the NRF2 pathway can restore the function and anti-tumour efficacy of NK cells, while blocking ferroptosis helps NK cells survive in the tumour (148, 151). In general, these observations show that NK cells are particularly susceptible to ferroptosis in the TME.

In short, these results highlight the complex and situationally dependent effects of ferroptosis on different inherent immune cell groups in the TME. Immunosuppressive M2 tumour-related macrophages and tumour-infiltrated neutrophils seem to be particularly sensitive, and natural killer cells also show significant susceptibility. On the contrary, medullary inhibitory cells (MDSCs) show different degrees of resistance or susceptibility according to the specific conditions of the TME. A more comprehensive understanding of how ferroptosis interacts with innate immune cells in the TME is crucial to the rational development and improvement of cancer immunotherapy.

### 4.3 Ferroptosis as a modulator of adaptive antitumor immune responses

Adaptive immune cells, especially B cells and T lymphocytes, will undergo functional changes in the TME. These changes enable them to further regulate the development of tumours (152, 153). The mechanism is rooted in the iron-deficient cancer cell secretion group, in which the components (including lipid peroxide) will destroy the function of effective immune cells and weaken their ability to identify tumour antigens. Similar to inherent immune cells, the adaptive immune cell group itself is inherently susceptible to ferroptosis, but this sensitivity also depends on the specific biological background.

B cells are very important for anti-tumour immunity, mainly by producing antibodies and regulating T cell responses (154). It is worth noting that congenital B-like cells, including B1 cells and marginal B cells, have particularly active lipid metabolism and rely on GPX4 to maintain their antibody production and overall function. Therefore, when GPX4 is missing, these cells are highly sensitive to ferroptosis (155). Although the data shows that targeting GPX4 may become a feasible strategy for specific B cell malignant tumours, comprehensive research is still needed to fully understand the extent to which ferroptosis affects the anti-tumour immune function of B cell dependence.

Many studies have shown that under various experimental conditions, T cells show considerable resistance to ferroptosis, which means that ferroptosis in cancer cells can be specifically induced without compromising the anti-tumour response mediated by T cells. Preclinical data demonstrate that genetic ablation of Slc7a11 in murine models suppresses tumor expansion without compromising T cell proliferation or function. Consequently, this intervention has been shown to potentiate the therapeutic efficacy of immune checkpoint blockade (156). Similarly, ferroptosis induced by cysteine deprivation or supplementation of arachidonic acid supports (and even enhances) the proliferation and effect function of T cells in the TME while limiting tumour growth. On the contrary, inhibiting ACSL4-driven ferroptosis will weaken the anti-tumour activity mediated by T cells (120, 121).

Inhibiting GPX4 by chemical inhibition or gene knockout can promote an increase in the number of CD4+ and CD8+ T cell groups in the three-negative breast cancer mouse model (139). In addition, it is reported that a variety of nanoparticle-mediated ferroptosis induction methods can also increase the infiltration of T cells into tumours (157, 158). In short, these research results show that in many cases, inducing ferroptosis does not impair the viability of T cells, but will enhance the anti-tumour immune response mediated by T cells (159, 160).

Contrary to previous reports that T cells can generally resist ferroptosis, some studies have shown that inhibiting GPX4 can make T cells very vulnerable. In the model of specific knockout of GPX4 in T cells, these cells will quickly accumulate lipid peroxide in their membranes and cause ferroptosis (161). Notably, CD8+ T lymphocytes exhibit greater vulnerability to ferroptosis induced by GPX4 inhibition compared to malignant cells (162). Elevated CD36 expression in tumor-infiltrating CD8+ T cells, which facilitates fatty acid uptake, exacerbates lipid peroxidation and ferroptosis, thereby diminishing their overall anti-tumor efficacy. It is noteworthy that the function of CD8+ T cells can be saved by CD36 gene knockout or drug-induced ferroptosis inhibition (163, 164).

The GPX4-mediated ferroptosis protection is crucial for CD4+ T cells. Regulating T cells (Treg cells), which are famous for their inherent immunosuppressive activity and inhibition of anti-tumour immune response, show significant resistance to ferroptosis, which is likely due to their high GPX4 levels. Selective deletion of GPX4 in Treg cells will cause ferroptosis, which will lead to enhanced anti-tumour immunity (165, 166). Similarly, the survival and function of CD4+ T cell subgroups that promote anti-tumour immunity - follicular-assisted T cells (T_FH cells) also depend on GPX4 to maintain, although the specific pathways of controlling the ferroptosis of T_FH cells in the TME have not been fully clarified (167).

In general, these research results show that triggering ferroptosis by missing SLC7A11 or GPX4 can differentially affect the function of different T cell subgroups. T cells exhibit a marked dependence on GPX4 rather than SLC7A11. This reliance likely reflects the low abundance and minimal functional contribution of SLC7A11 within this lineage (168, 169). By inhibiting SLC7A11 or supplementing arachidonic acid-triggered ferroptosis, the anti-tumour response driven by CD8+ T cells can be maintained and in some cases enhanced. In contrast, inhibiting GPX4 produces different results according to different T cell subgroups: triggering ferroptosis in immunosuppressive Treg cells will enhance the anti-tumour immune response, while inhibiting GPX4 in CD8+ or T_FH cells will impair their anti-tumour function.

Together, these observations at the level of adaptive immunity illustrate that the immunological consequences of ferroptosis depend not only on the pathway being targeted, but also on the specific immune cell subsets involved.

Extending beyond adaptive immune cells, ferroptosis within the tumor microenvironment affects tumor cells as well as multiple innate immune populations, resulting in divergent immunological outcomes. Ferroptosis in tumor cells is frequently accompanied by lipid peroxidation and the release of stress-associated signals, which can support antigen presentation and promote immune cell recruitment under certain conditions (120, 139, 157, 170, 171). In contrast, when ferroptotic stress directly impacts immune effector cells, including macrophages, neutrophils, or natural killer cells, their viability and functional state may be altered in ways that either relieve or reinforce local immunosuppression (137, 143, 148, 149).

Accumulating evidence indicates that these opposing effects are shaped by the cellular identity of the affected population and the local metabolic environment. Immunosuppressive myeloid cells, such as M2-polarized macrophages, often display higher sensitivity to ferroptosis than their pro-inflammatory counterparts, suggesting a potential opportunity to selectively remodel the immune microenvironment (137, 140, 141, 172). However, ferroptosis has also been reported to promote the recruitment or functional polarization of myeloid cells toward immunosuppressive states in specific tumor contexts, underscoring that its immunological impact is not uniformly beneficial (143, 144).

Taken together, ferroptosis should be regarded as a context-dependent regulator of antitumor immunity within the tumor microenvironment rather than a uniformly immunostimulatory or immunosuppressive process. Its biological and therapeutic effects are determined by the cell types involved, the intensity and duration of ferroptotic stress, and the surrounding metabolic and inflammatory conditions (148, 149). In thyroid cancer, where long-term disease control is often required, these considerations highlight the importance of precisely modulating ferroptosis to suppress tumor growth while preserving protective immune function.

## 5 The impact of ferroptosis on thyroid cancer progression

**Figure 4:**
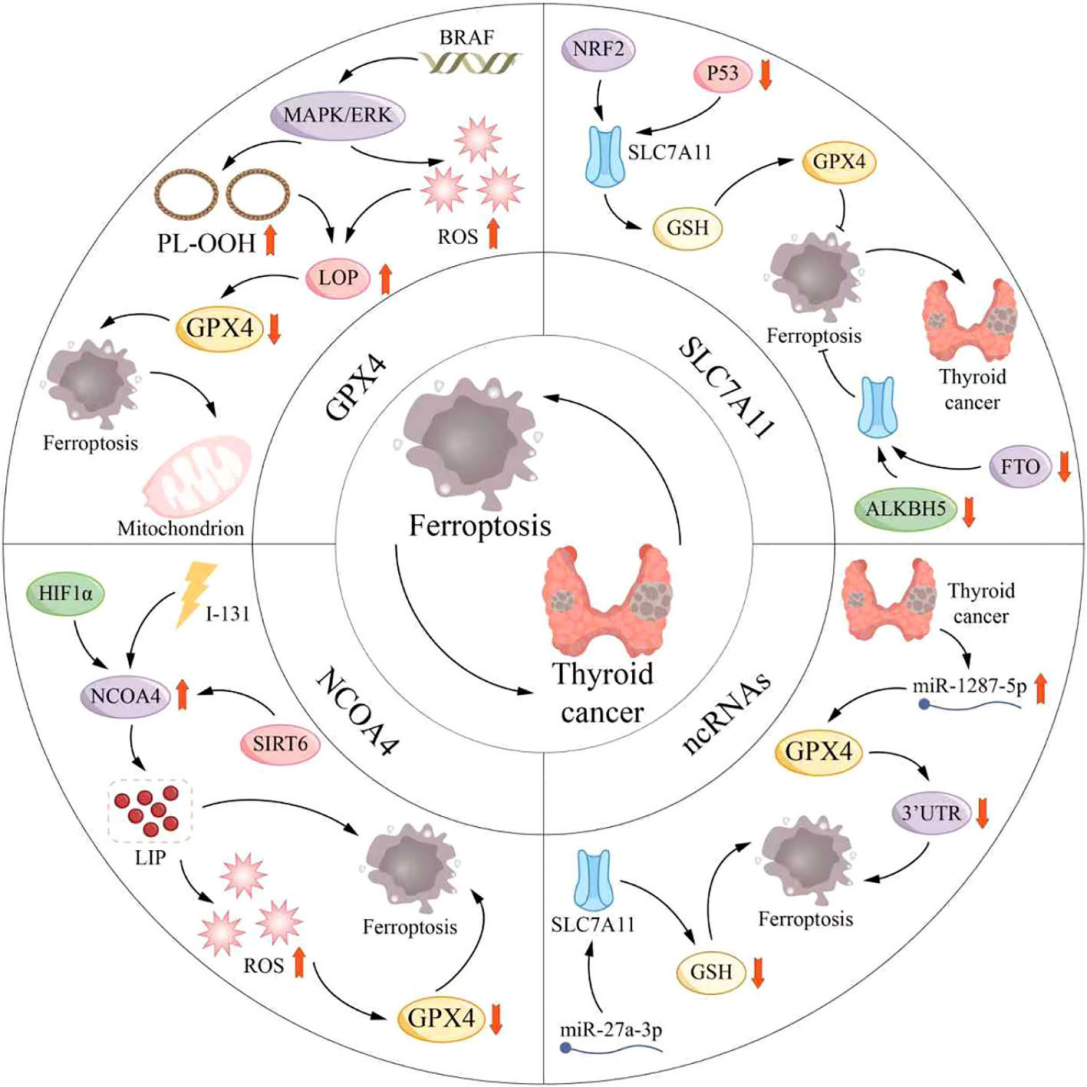
Role of ferroptosis in thyroid cancer. Ferroptosis is intricately linked to both the onset and advancement of tumors, as well as being crucial in the initiation and progression of thyroid cancer. Key mechanisms involved are Glutathione Peroxidase 4 (GPX4), Solute Carrier Family 7 Member 11 (SLC7A11), Nuclear Receptor Coactivator 4 (NCOA4), and non-coding RNAs (ncRNAs). BRAF, B-Raf proto-oncogene, serine/threonine kinase; ERK, Extracellular Signal-Regulated Kinase; HIF1α, Hypoxia-Inducible Factor 1 Alpha; LOP, Lipid Peroxide; MAPK, Mitogen-Activated Protein Kinase; NRF2, Nuclear Factor Erythroid 2-Related Factor 2; PL-OOH, Phospholipid Hydroperoxide; ROS, Reactive Oxygen Species; SIRT6, Sirtuin 6.

Emerging evidence implicates ferroptosis in multiple stages of malignancy, including tumorigenesis, metastatic spread, and the development of therapeutic resistance. Therefore, exploring the role of ferroptosis in cancer, including thyroid malignant tumours, has become an important research focus, involving a series of molecular mechanisms and potential treatments. Contemporary investigations center on modulating ferroptosis via specific signaling cascades, offering novel perspectives for thyroid cancer therapy (**Figure 4**). Ferritin deficiency exhibits a strong correlation with both tumor initiation and disease advancement, exerting significant influence on the progression of thyroid carcinoma, a relationship elaborated upon in a subsequent section.

### 5.1 GPX4

As a unique intracellular selenium protein, GPX4 uses glutathione as a reducing agent to prevent lipid peroxide from accumulating in cells, which is crucial to maintaining cell redox balance and preventing oxidative damage (173). Unlike other GPX family members, GPX4 is relatively low in dependence on glutathione and can directly remove lipid peroxides on the membrane, which makes it the core regulatory factor of ferroptosis (174). In thyroid cancer tissue, the expression of GPX4 is significantly increased, and its overexpression is closely related to the enhancement of tumour invasion and the poor prognosis of patients (175). Clinical research confirms that the overall survival rate of patients with high expression of GPX4 is significantly lower than that of patients with baseline expression level (176).

Mechanistically, high GPX4 expression safeguards thyroid carcinoma from both ferroptosis and metastatic dissemination. Conversely, reducing or ablating GPX4 triggers the accumulation of ROS, depletes glutathione (GSH) reserves, and precipitates ferroptotic cell demise, thereby diminishing cellular viability (175). Whether it is genetic intervention or the use of pharmacological inhibitors such as RSL3 and diaryl ether compounds, it can effectively target GPX4, thus inducing ferroptosis, impairing mitochondrial function, inhibiting mTOR pathways, enhancing DNA damage and reducing the migration of thyroid cancer cells (176). The sensitivity of thyroid cancer cells to GPX4 inhibition is heterogeneous, which mainly depends on their genetic background - especially the presence or absence of BRAF, RAS, PIK3CA gene mutations and TERT promoter mutations. This susceptibility of mutation dependence has been confirmed in experiments in traditional single-layer culture and three-dimensional sphere systems (177). In short, these evidences emphasise that GPX4 is a core determinant of ferroptosis control and the development of thyroid cancer, and its inhibition is positioned as a potential treatment development direction in the clinical environment.

### 5.2 SLC7A11

As a component of the Xc− reverse transporter, the transmembrane protein SLC7A11 is responsible for the exchange of cysteine and glutamic acid, and is a key inhibitor of ferroptosis (178, 179). Its expression is significantly increased in thyroid cancer, promotes tumour proliferation by inhibiting ferroptosis, and is associated with poor prognosis (180). Diverse molecular mediators influence ferroptosis in thyroid malignancies through the specific modulation of SLC7A11. For example, cyclic RNAs (circRNAs) such as circ-0067934 and circ-0008274 can enhance the expression of SLC7A11, thus promoting malignant progression; meanwhile, ETS variant transcription factor 4 (ETV4) has been proven to upregulate SLC7A11 *in vitro* and *in vivo*, which in turn promotes the proliferation and migration of cancer cells (181, 182). On the contrary, tumour inhibitory genes and epigenetic regulatory factors enhance the sensitivity of cells to ferroptosis by inhibiting the activity of SLC7A11. Tumour inhibitor p53 effectively limits the progression of thyroid cancer by inhibiting the transcription of SLC7A11 and promoting the occurrence of ferroptosis (183).

Likewise, BAP1 limits glutathione levels by suppressing SLC7A11 expression. This inhibition facilitates lipid peroxide buildup, thereby precipitating ferroptosis (184). In the epigenetic mechanism, m6A RNA methylation has become a key determinant of thyroid tumour biology. In thyroid papillary carcinoma, the reduced activity of demethylase ALKBH5 and FTO will change cell homeostasis, which is conducive to malignant growth. Experimental reactivation of these enzymes can offset this effect by reprogramming the expression of SLC7A11 and restoring the susceptibility to ferroptosis of cells, thus inhibiting tumour expansion (180). In terms of mechanism, ALKBH5 promotes ferroptosis through the ALKBH5–TIAM1–Nrf2/HO-1 axis, while FTO reduces the expression of SLC7A11 through the regulation of m6A dependence, thus increasing the levels of ROS and Fe²+ (20). In general, these research findings highlight that SLC7A11, as the core hub of ferroptosis regulation and an attractive therapeutic target for thyroid cancer, is subject to multiple regulation of transcriptional, post-transcriptional and epigenetic mechanisms, providing multiple ways for clinical intervention.

### 5.3 Nuclear receptor coactivator 4

The ferritin autophagy process is a key link in cells to release iron, increase unstable iron pools and therefore be more sensitive to ferroptosis, in which NCOA4 acts as a cargo receptor (185). Therapeutic activation of this pathway has been demonstrated to suppress thyroid tumor progression. For instance, vitamin C induces ferroptosis by triggering ferritinophagy. In a parallel mechanism, SIRT6 potentiates NCOA4-dependent ferritinophagy, a process that expands the intracellular labile iron pool and renders cells vulnerable to ferroptosis (186, 187). *In vivo* research evidence further supports this mechanism. The use of ferroptosis-inducing agent sulfopyridine can effectively inhibit tumour growth driven by SIRT6 upregulation (187). These findings show that the SIRT6–NCOA4–ferrin autophagy axis is the core regulatory node of ferroptosis, and also provides a promising pathway for the treatment intervention of thyroid cancer.

### 5.4 Non-coding RNAs

In oncology, non-coding RNAs act as fundamental regulators of gene expression and cellular signaling networks. The most extensively researched subclasses include microRNAs (miRNAs), long non-coding RNAs (lncRNAs), and circular RNAs (circRNAs) (188, 189). These molecules involve almost all aspects of tumour biology, including pathogenesis, diagnostic evaluation, prognostic judgement and treatment response (190, 191). The capacity of non-coding RNAs to modulate ferroptosis is increasingly acknowledged, establishing these molecules as central mediators and promising therapeutic targets in thyroid cancer progression. Among these, Long noncoding RNAs (lncRNAs) are transcripts defined as being longer than 200 nucleotides. Through interaction with DNA, RNA or proteins, they play a regulatory role at multiple levels such as chromatin dynamics, transcription program, RNA processing, stability, translation and competitive endogenous RNA (ceRNA) networks (192, 193).

In thyroid papillary carcinoma, the high expression of long-chain non-coding RNA CERS6-AS1 and LASP1 was associated with adverse clinical outcomes, while miR-497-5p showed the opposite expression pattern (194). In terms of mechanism, inhibiting CERS6-AS1 will reduce the vitality of cancer cells and enhance their susceptibility to ferroptosis by regulating the miR-497-5p/LASP1 axis (194). In contrast, circular RNAs (circRNAs) are characterized by a covalently closed, single-stranded architecture that confers exceptional stability and tissue-specific distribution. These cyclic RNA molecules can play a variety of functions such as transcription regulatory factors, miRNA sponges or protein scaffolds in the TME (195, 196). CircKIF4A has been recorded in a variety of malignant tumours, including thyroid cancer. It regulates GPX4 activity by adsorbing miR-1231, thus inhibiting the occurrence of ferroptosis and promoting tumour proliferation and migration (197, 198). Similarly, circ_0067934 adsorbs miR-545-3p through sponge action, thus driving the overexpression of SLC7A11, thus enhancing tumour cell proliferation and protecting cells from ferroptosis (199).

In short, these studies together reveal a precise regulatory network driven by non-coding RNA, which coordinates the process of ferroptosis in thyroid cancer, and also highlights the broad prospect of non-coding RNA as a potentially feasible target for diagnostic markers and therapeutic interventions.

### 5.5 Emerging role of nanomedicine in ferroptosis regulation

In addition to intrinsic molecular regulators, increasing attention has been directed toward nanomedicine-based strategies as modulators of ferroptosis, thereby providing new perspectives on thyroid cancer progression. Accumulating evidence indicates that engineered nanoparticles can interfere with intracellular iron homeostasis, promote reactive oxygen species (ROS) generation, and intensify lipid peroxidation cascades, all of which converge on the execution of ferroptotic cell death (200). Such nano-enabled amplification of oxidative stress is particularly relevant to ferroptosis, given its dependence on iron-catalyzed lipid peroxide accumulation and redox imbalance.

Beyond directly perturbing redox and iron metabolism, nanomedicine platforms also offer unique advantages in the controlled delivery of ferroptosis-related cues. By improving pharmacokinetics, tumor accumulation, and intracellular release of ferroptosis-inducing agents, nanoformulations may enhance tumor selectivity while reducing systemic exposure and off-target toxicity (200, 201). In this context, nanocarrier-based systems have been proposed as effective tools to potentiate oxidative stress–dependent, non-apoptotic cell death pathways in cancer cells, particularly in tumors exhibiting resistance to conventional therapies.

Although direct evidence in thyroid cancer remains limited, these advances collectively suggest that nanomedicine-assisted regulation of ferroptosis represents a promising adjunct strategy to influence tumor behavior. Further investigation using thyroid cancer–specific models will be required to clarify how nano-enabled ferroptosis modulation may be integrated into disease progression studies and future therapeutic frameworks.

Despite these promising features, several major translational challenges remain. First, systemic safety is a central concern, because ferroptosis induction is inherently linked to disruption of redox buffering and iron–lipid homeostasis, which are also essential for normal tissue integrity. Reviews in the nanomedicine–ferroptosis field have emphasized that the same nano-enabled amplification of oxidative stress that is therapeutically desirable in tumors may, if not sufficiently confined, translate into dose-limiting toxicities, particularly in metabolically active organs that are prone to oxidative injury (e.g., liver and kidney) or highly sensitive to lipid peroxidation–driven damage (e.g., nervous system) (202, 203). Second, achieving truly tumor-selective ferroptosis remains nontrivial. Even when nanoformulations improve pharmacokinetics, heterogeneous vascular permeability, stromal barriers, and variable cellular uptake can lead to inconsistent intratumoral deposition. Under such conditions, incomplete tumor-specific accumulation may increase systemic exposure and enable off-target lipid peroxidation and ferroptosis-like injury in normal tissues. This concern is particularly relevant for ferroptosis-triggering nanomaterials whose physicochemical properties (e.g., surface chemistry and catalytic activity) can strongly influence ferroptotic potency and toxicity risk, highlighting the need for rigorous structure–activity and safety profiling before clinical translation (203, 204). Third, the field still lacks reliable, non-invasive biomarkers to monitor ferroptosis engagement in patients, which complicates both efficacy assessment and real-time safety surveillance during early-phase trials. Current ferroptosis readouts largely rely on tissue-based molecular or biochemical measurements, limiting their feasibility for longitudinal monitoring. Recent work has begun to explore imaging-oriented solutions, including system Xc^-^–linked PET tracer approaches for tracking ferroptosis-related target engagement *in vivo*, as well as ferroptosis-associated MRI strategies that can sensitively detect ferroptosis-linked organ injuries (205, 206). However, these methods remain in early development and require further validation, standardization, and clinical correlation before they can serve as routine trial endpoints.

Taken together, these considerations suggest that while nanomedicine may enhance delivery and efficacy of ferroptosis-based interventions, clinical feasibility will likely depend on simultaneously improving tumor selectivity, defining a safety window that minimizes systemic and off-target ferroptotic damage, and developing practical non-invasive monitoring tools to guide dosing and patient selection.

Thyroid cancer includes distinct subtypes, such as PTC, FTC, ATC, and MTC. Current studies on ferroptosis in thyroid cancer are unevenly distributed across these subtypes, and direct comparative analyses remain limited. Most mechanistic investigations of ferroptosis-related regulators, including GPX4, SLC7A11, and iron metabolism–associated pathways, have focused on PTC, whereas data from FTC, ATC, and MTC are relatively scarce (207–209).

With respect to iron metabolism, transferrin receptor 1 (TFRC/CD71), a key mediator of cellular iron uptake, has been examined in thyroid cancer with inconsistent results (207–210). Some studies have reported increased TFRC expression in thyroid cancer tissues compared with normal thyroid tissue (208, 210), whereas others have shown TFRC downregulation or no significant difference in PTC (207, 209). These findings indicate that TFRC expression in thyroid cancer remains controversial, and whether such differences are related to tumor stage or subtype has not been systematically evaluated. At the cellular level, ATC cell lines such as 8505C have been reported to exhibit relatively low CD71 expression and increased tolerance to iron overload and ferroptosis-related stress (187). In contrast, FTC-derived cells, including FTC-133, show higher sensitivity to ferroptosis induction under certain experimental conditions (187).

Differences among subtypes have also been observed in ferroptosis-related regulatory pathways. In ATC models, overexpression of SIRT6 has been shown to upregulate NCOA4 expression, increase intracellular Fe²^+^ levels, and suppress tumor cell growth (187), indicating that ferritinophagy-associated iron release participates in ferroptosis regulation in aggressive thyroid cancer. By comparison, in PTC models, overexpression of the fat mass and obesity-associated protein (FTO) has been reported to downregulate SLC7A11, induce ferroptosis, and inhibit tumor formation (180). These findings indicate that ferroptosis-related regulators involved in different thyroid cancer subtypes are not identical.

However, current evidence remains limited and fragmented, making it difficult to draw definitive conclusions regarding subtype-specific differences in ferroptosis regulation in thyroid cancer. Further clarification of ferroptosis heterogeneity will require more refined analyses integrating tumor subtype, iron metabolism, and ferroptosis regulation.

## 6 Diagnosis and assessment of ferroptosis in thyroid cancer

The assessment of thyroid cancer, encompassing both diagnosis and prognosis, hinges on a combination of imaging modalities, histological examination, and serological markers. Histopathological assessment remains the definitive criterion for thyroid cancer diagnosis, supported by cell morphology, histological grading, evaluation of thyroid capsule integrity, and ancillary functional assays (211, 212).

Some thyroid malignant tumours have a high risk of lymphatic metastasis and a tendency to relapse, which seriously affects the survival rate of patients and highlights the need for early prognostic evaluation and timely clinical intervention. Recent research shows that genes associated with ferroptosis may become valuable biomarkers for predicting the clinical outcome of thyroid cancer. The wide application of bioinformatics methods enables researchers to deeply analyse the relationship between gene expression patterns and tumour prognosis with the help of public databases and proprietary sequencing data (213, 214). In addition to simple prognostic prediction, these comprehensive analyses have also contributed to the construction of multi-factor predictive feature models, which not only improve the accuracy of prognostic judgement, but also provide valuable insights for understanding the tumour immune microenvironment, drug resistance, treatment guidance and disease monitoring (215). In order to improve the prognostic assessment and provide more comprehensive insights into the TME and treatment options, a variety of ferroptosis-related genetic characteristic models have been proposed.

GPX4 is the central inhibitor of ferroptosis; although its down-regulation can trigger the death of ferroptotic cell death and inhibit tumour growth, it is commonly upregulated in thyroid cancer, which is correlated with advanced disease, invasive pathological characteristics and poor overall survival, which makes it a powerful prognostic biomarker (96, 216). Similarly, NADP(H) oxidoreductase AKR1C3 is widely regarded as a negative regulatory factor for ferroptosis (217). High expression of AKR1C3 in thyroid cancer leads to drug resistance and poor prognosis, while inhibiting its expression can inhibit tumour growth (218). G protein-coupled receptor DRD4 regulates the chemotherapy sensitivity of cells by inhibiting ferroptosis (219). In thyroid cancer, the overexpression of DRD4 is associated with higher risk scores and worse outcomes, highlighting its potential as a prognostic indicator (209).

Other genes, including SRXN1, HMGCR and DPP4, further demonstrate the complexity of ferroptosis regulation in thyroid cancer (220, 221). SRXN1 promotes tumour migration and invasion by regulating reactive oxygen, and is associated with poor survival (222). HMGCR activates GPX4 through the methoxyvalproate pathway, thus inhibiting ferroptosis; its high expression has been observed in thyroid cancer and many other cancers, and has always been associated with poor prognosis (210). On the contrary, DPP4 plays a dual role: although some studies show that its high expression may reduce the risk of thyroid cancer (209), other studies link it with BRAF mutation, advanced tumour staging, and promoting proliferation through stable integin and TGF signal conduction, which indicates that its role is Dependence (223, 224). In addition, iron transport proteins such as TF and TFRC control ferroptosis by regulating iron intake (207, 209).

Although direct clinical evidence supporting the diagnostic or prognostic value of FSP1 in thyroid cancer remains limited, multiple solid tumor studies indicate that as an independent inhibitor of ferroptosis via glutathione, FSP1 correlates with tumor progression, poor prognosis, and therapeutic resistance. This provides a mechanistic basis for its potential significance in thyroid cancer (99). Mechanistically (225), FSP1 reduces oxidized coenzyme Q10 to its antioxidant form CoQ10H_2_ via the FSP1-coenzyme Q10-NAD(P)H axis, thereby scavenging lipid peroxyl radicals and inhibiting membrane lipid peroxidation. This pathway maintains ferroptosis resistance even when GPX4 activity is impaired. Furthermore, FSP1 participates in the non-classical vitamin K redox cycle and promotes ESCRT-III-mediated membrane repair, further enhancing cellular tolerance to ferroptosis-induced stress (226). In cancer research (227), FSP1 is upregulated in hepatocellular carcinoma and other malignancies, correlating with increased tumor burden, enhanced invasiveness, and reduced survival rates. Its expression is regulated by the KEAP1/NRF2 oxidative stress pathway, which is frequently activated in thyroid cancer, suggesting FSP1 may exert similar antioxidant and ferroptosis-inhibitory functions in this context. Overall, although specific validation in thyroid cancer remains necessary, FSP1’s central role in ferroptosis regulation supports its identification as a potential mechanistic factor and biomarker candidate for thyroid cancer.

Based on these insights, a variety of prognostic models have been developed using ferroptosis-related genes (FRGs) to improve the risk stratification and clinical prediction of thyroid cancer. Prognostic signatures comprising genes such as GPX4, AKR1C3, BID, FBXW7, and MAP3K5 (218), or alternative panels featuring DPP4, TYRO3, TIMP1, CDKN2A, SNCA, NR4A1, IL-6, and FABP4 (228), have demonstrated clinical utility. These models successfully stratify patients into distinct risk categories and function as independent predictors of overall survival. However, despite differences in gene composition and model construction strategies, most FRG-based prognostic models share a broadly similar statistical architecture relying on transcriptomic profiling and multivariate regression modelling. Importantly, these models are predominantly developed through retrospective bioinformatics analyses of publicly available datasets, particularly TCGA and GEO cohorts. While such approaches enable efficient discovery of ferroptosis-related prognostic signatures, they are inherently subject to dataset selection bias, limited clinical annotation, and variability in sample processing and sequencing platforms.

In contrast to ATA risk stratification, which directly informs postoperative management decisions such as the intensity of radioactive iodine therapy, TSH suppression targets, and follow-up intervals, FRG-based models primarily provide probabilistic survival estimates derived from transcriptomic patterns rather than actionable therapeutic guidance. Moreover, their predictive performance remains insufficiently validated in large-scale, independent, and prospective clinical cohorts, and their biological relevance at the protein and functional levels is still incompletely characterised. As a result, the current clinical value of FRG-based signatures lies mainly in biological risk characterization, mechanistic hypothesis generation, and exploratory patient stratification, rather than immediate treatment selection or guideline-level decision making.

Further evaluation of these models uncovers a significant association with the tumor immune microenvironment. Specifically, the data delineate variations in immune cell infiltration and the extent of engagement in anti-tumor signaling pathways (229). Recently, after recognising the dual importance of ferroptosis and non-coding RNA, a model based on long-chain non-coding RNA (lncRNA) has been constructed. A prominent example is the long-chain non-coding RNA prognostic scoring framework associated with ferroptosis (Ferr-LPM), in which the higher score is associated with the patient’s poor survival, lower tumour purity and higher immune checkpoint inhibitor treatment sensitivity (230). By combining gene regulation with immune microenvironmental characteristics, such models not only improve the accuracy of prognostic judgement, but also provide valuable tools for guiding treatment decision-making and monitoring the progress of the disease. Nevertheless, their current clinical translation remains constrained by shared challenges, including dependence on bulk RNA sequencing data, cohort-specific cut-off values for risk stratification, limited prospective or multicenter validation, and the lack of harmonization with established clinical decision frameworks, which collectively hinder immediate implementation in routine practice.

## 7 Ferroptosis in thyroid cancer: therapeutic landscape and opportunities

### 7.1 Clinical evidence

Standard therapeutic management for thyroid cancer comprises surgical extirpation, succeeded by radioiodine ablation and suppression of TSH levels (12). For papillary thyroid cancer in the thyroid gland, especially tumours with a diameter of 1 to 4 cm, the best surgical scope has always been controversial. Current protocols from the American Thyroid Association (ATA) and the National Comprehensive Cancer Network (NCCN) advocate for risk-adapted surgical strategies. While total thyroidectomy is mandated for patients with high-risk features, thyroid lobectomy remains a viable alternative for specific low-risk cohorts (231, 232). Although early studies favoured total thyroidectomy based on the belief that it could improve the survival rate, recent evidence shows that, after taking into account risk factors, lobectomy and total thyroidectomy are comparable in terms of recurrence and survival outcomes (233, 234). Lobectomy has the advantages of thyroidectomy, including fewer complications, hypoparathyroidism and reduced risk of laryngeal nerve damage, and the possibility of maintaining normal thyroid function, although it has limitations in postoperative monitoring and recurrence detection (235, 236). Therefore, surgical decisions ought to be guided by the patient’s individual factors, including multifocal tumour, contralateral nodules, anaesthesia risk, quality of life considerations, and the expected degree of dependence on thyroid hormone treatment.

In differentiated thyroid cancer, the application of radioactive iodine has evolved into a risk stratification strategy, which is divided into residual tissue ablation, auxiliary intervention and targeted treatment of residual or recurrent diseases according to its use (231). According to the current guidelines of the ATA, radioiodine treatment is mainly recommended for patients with medium and high-risk characteristics, while those who are classified as low-risk and in good postoperative condition usually benefit little from it (231). Current literature bolsters the recommendation to employ lower radioactive iodine doses (e.g., 30 mCi) to mitigate systemic toxicity and enhance patient well-being (237, 238). Therefore, in the past two decades, the application of radioiodine in limited thyroid cancer has decreased (239). Efficacy monitoring largely depends on serum thyroglobulin (Tg) levels and imaging evaluation; however, for patients with anti-thyroglobulin antibodies in the body or patients after partial thyroidectomy, the reliability of this monitoring is reduced, which highlights the replacement biomarkers and personalised follow-up the demand for visiting strategies (12).

Thyroid hormone suppression therapy aims to mitigate the mitogenic effect of TSH on thyroid cancer cells; consequently, its clinical deployment has evolved toward increased selectivity and individualization. Early studies supported its universal use, but later evidence showed that the benefits were limited for low-risk or long-term remission patients, and its main advantage was in patients with medium and high risk or disease activity (240, 241). Concerns about side effects such as cardiovascular complications and osteoporosis, as well as emerging and even questioning data on its efficacy in invasive cancer, further narrowed its indications (242, 243). Future prospective studies may need to combine tumour genotypes to more accurately define which patients can really benefit from thyroid-stimulating hormone inhibitory therapy.

Although ferroptosis has shown potential in thyroid cancer treatment, several obstacles limit its clinical translation. First, selective delivery of ferroptosis inducers to thyroid cancer tissue and/or metastatic lesions remains a major challenge. Thyroid cancer frequently involves cervical lymph nodes and may metastasize to distant organs such as the lung and bone. Nanomedicine-based approaches have been explored to improve drug delivery efficiency and reduce off-target exposure (200); however, current evidence is largely derived from *in vitro* or animal studies, and their clinical feasibility in thyroid cancer has yet to be established. Second, systemic toxicity associated with ferroptosis induction cannot be overlooked (244). Ferroptosis is not restricted to malignant cells, and normal tissues such as the liver and kidney are also sensitive to iron imbalance and lipid peroxidation. Non-selective induction of ferroptosis may therefore cause off-target tissue injury, a concern that becomes more relevant in the setting of long-term treatment or combination therapy. Third, reliable and clinically applicable methods for monitoring ferroptosis in humans are still lacking. Current evaluation of ferroptosis mainly relies on the expression of key regulatory molecules in tumor tissues (such as GPX4, SLC7A11, and FSP1), as well as indicators related to lipid peroxidation and iron metabolism. While these approaches are useful for mechanistic studies and tissue-level assessment, they are largely based on tissue samples or experimental models and do not meet clinical requirements for non-invasive, repeatable, and dynamic monitoring. In addition, lipid peroxidation and iron metabolic status in humans are highly dynamic and influenced by inflammation, metabolic conditions, and concomitant treatments, which further limits their specificity and stability as clinical indicators. Consequently, translating current molecular and tissue-based assessment strategies into tools suitable for patient follow-up and treatment response evaluation remains a significant barrier to the clinical application of ferroptosis-based approaches in thyroid cancer.

### 7.2 Preclinical evidence

Although progress has been made in surgical resection, radioiodine treatment and thyroid-stimulating hormone inhibition therapy, a considerable number of thyroid cancer patients (nearly 20%) eventually experience disease recurrence or have treatment resistance, which significantly affects the long-term survival outcome (12, 13). These limitations highlight the urgent need to go beyond conventional programs and develop innovative treatment strategies (245). Experimental evidence shows that regulating ferroptosis-related factors such as GPX4, FSP1 and GCH1 can overcome the drug resistance mechanism and inhibit tumour progression (246). Although this strategy of targeting ferroptosis has been widely studied in highly invasive malignant tumours, its application in thyroid cancer is still insufficient, so the research attention has been relatively limited. Given the persistent difficulties in managing refractory and recurrent malignancy, the ferroptosis pathway offers a viable therapeutic target for enhancing outcomes in thyroid cancer patients.

Accumulating data indicate that specific natural products and synthetic small molecules can suppress thyroid cancer progression by triggering ferroptosis. Among many natural compounds, curcumin is a bioactive polyphenol extracted from the rhizome of turmeric, which is traditionally used as a pigment and flavouring agent. It has received more and more attention because it shows broad-spectrum anti-cancer activity in malignant tumours of the mammary gland, liver, lung and stomach (247, 248). In terms of mechanism, curcumin can induce reactive oxygen (ROS) surge and accelerate lipid peroxidation. These effects are driven by simultaneously upregulating heme oxygenase-1 (HO-1) and inhibiting GPX4 activity (249, 250). In thyroid cancer, these coordinated molecular changes not only enhance oxidative stress, but also promote the interaction between HO-1 and GPX4, eventually pushing the redox balance to ferroptosis (251). By using this iron-promoting death mechanism, curcurmin can effectively hinder the survival and progression of tumour cells, which highlights its potential as a natural compound associated with the treatment of thyroid cancer.

Vitamin C is not only an important nutrient to promote iron absorption, but also can effectively regulate the process of ferroptosis in thyroid cancer (252). In addition to known effects such as immune regulation, vitamin C promotes ferritin to release free iron through autophagy, and these iron ions then produce a large amount of reactive oxygen through the Fenton reaction. The continuous accumulation of reactive oxygen continues to promote the lipid peroxidation reaction, and finally induces ferroptosis (253). Active oxygen continues to accumulate, continuously promoting lipid peroxidation reaction, and finally inducing ferroptosis (19). In undifferentiated thyroid carcinoma models, vitamin C administration markedly curtails tumor cell proliferation by instigating the ferroptosis pathway. In short, these research findings highlight that vitamin C, as a promising preparation, can use ferroptosis to therapeutically inhibit invasive thyroid cancer (19).

Neferine, a bisbenzylisoquinoline alkaloid isolated from the seed embryo of Nelumbo nucifera, possesses diverse medicinal properties, including anticancer, anti-inflammatory, antioxidant, and cardioprotective activities (254, 255). In thyroid cancer, studies have reported that neferine can induce ferroptosis through multiple mechanisms (256). Specifically, neferine increases intracellular iron levels and reactive oxygen species while downregulating key ferroptosis regulators, such as SLC7A11 and GPX4, thereby disrupting cellular redox homeostasis. In addition, neferine promotes apoptosis in thyroid cancer cell lines, including IHH-4 and CAL-62, and suppresses the Nrf2/HO-1/NQO1 antioxidant signaling pathway, further sensitizing tumor cells to ferroptosis. These effects have also been validated in animal models, in which neferine treatment effectively promotes tumor cell death and inhibits thyroid cancer progression *in vivo* (256). In short, these findings indicate that neferine is a promising natural compound targeting ferroptosis for thyroid cancer treatment.

In addition to natural products, synthetic derivatives also show hope; for example, the dihydroisoxazole derivative DE16 inhibits mitochondrial polarisation and down-regulates GPX4, imitates the role of classical de-iron inducers, and exerts a strong anti-tumour effect in thyroid cancer (257). It is noteworthy that even clinically useable targeted drugs, such as anlotinib, are associated with the regulation of ferroptosis.

Anlotinib is a multi-target small molecule tyrosine kinase inhibitor, which has shown strong anti-angiogenic activity in thyroid cancer. Recent studies have extended its mechanism of action beyond angiogenesis, revealing its key role in regulating ferroptosis. Anlotinib administration downregulates key ferroptosis-associated proteins, including transferrin, ferritin light chain (FTL), and GPX4. Conversely, it amplifies oxidative stress by elevating ROS and lipid peroxidation (258, 259). Markers for apoptosis, pyroptosis, and necroptosis remained unaltered, identifying ferroptosis as the primary mechanism mediating this anti-tumor response. The study of the mechanism further shows that anlotinib can induce protective autophagy, which can fight the ferroptosis. On the contrary, blocking autophagy will enhance the ferroptosis effect of anlotinib and enhance its tumour inhibition effect in *in vitro* and *in vivo* models (259).

The BRAFV600E mutation is one of the most common carcinogens in thyroid papillary carcinoma, but the treatment of tumours carrying this mutation is increasingly challenged by the development of treatment resistance (260). In order to meet this challenge, recent studies have explored strategies that transcend the inhibition of classical kinase. It is worth noting that a compound based on diaryl ether was designed to utilise the ferroptosis pathway in thyroid cancer cells (257). The molecule induces lipid peroxidation-mediated cell death, rather than traditional cell apoptosis, by inhibiting the expression of GPX4 and destroying the polarisation stability of mitochondria. Through the coordinated modulation of redox homeostasis and mitochondrial function, these compounds demonstrate potent anti-tumor efficacy. These findings position ferroptosis induction as a viable therapeutic avenue for BRAF V600E-mutant thyroid cancer.

Overall, although accumulating preclinical studies suggest that ferroptosis may represent a potential therapeutic vulnerability across multiple thyroid cancer subtypes, the current body of evidence remains largely confined to mechanistic exploration, and its overall translational maturity is clearly insufficient (see **Table 1**). Most available studies rely heavily on *in vitro* culture systems and simplified xenograft models, which are typically derived from a limited number of thyroid cancer cell lines and therefore fail to adequately capture the pronounced heterogeneity observed in patients, particularly with respect to metabolic states, tumor–stroma interactions, and immune microenvironmental features. Consequently, whether ferroptosis sensitivity observed under experimental conditions can be extrapolated to more differentiated tumors or clinically complex disease contexts remains unsupported by direct evidence.

**Table 1:** Ferroptosis-targeting strategies and experimental supporting evidence in thyroid cancer.

Intervention	Model	Mechanism	Extent of mechanistic validation	Translational limitation	References
Curcumin	FTC-133 cell and FTC-238 cell	GPX4↓, HO-1↑	In vitro phenotypic and pathway-associated validation	Evidence is largely limited to in vitro models, with context-dependent roles of HO-1 and unclear in vivo relevance	(251)
Vitamin C	8505C cell and C643 cell	GPX4↓, PTGS↑	In vitro phenotypic and ferritinophagy pathway validation	Evidence is limited to in vitro ATC models, and clinical relevance of high-dose vitamin C remains unclear	(19)
Neferine	IHH-4 and CAL-62 cell lines;IHH-4-derived xenografts in BALB/c nude mice	Nrf2/HO-1/NQO1↓	In vitro functional validation with xenograft tumor model confirmation	Although supported by xenograft models, the study relies on a single tumor model and lacks pharmacokinetic and safety assessment	(256)
Tenacissoside H	8505C cell	GPX4↓, xCT↓, HO–1↓, TFR↓	In vitro functional and pathway-associated validation	Mechanisms remain incompletely defined, and in vivo toxicity is unassessed	(262)
Anlotinib	8505 C cell and KHM-5 M cell	ER stress–mediated ferroptosis (PERK/eIF2α/ATF4↑/GPX4↓/ROS↑)	In vitro pharmacologic and pathway-associated validation	Preclinical evidence only; clinical relevance remains unclear	(277)
	KHM-5M, C643, 8505C and TPC-1 cell lines;8505C-derived xenografts in BALB/c nude mice	Autophagy-associated ferroptosis	In vitro functional validation with xenograft tumor model confirmation	Limited to preclinical ATC models; clinical validation is lacking	(259)
DE16(the diaryl ether derivative 16)	MDA-T32 cell and MDA-T41 cell	GPX4↓	In vitro pharmacologic target-specific validation	Preclinical only (cells + in silico); no in vivo/clinical validation.	(257)
Isobavachalcone + doxorubicin	ATC cells, xenograft	ROS↑, GSH↓, MDA↑ / ferroptosis↑	In vitro functional validation with xenograft tumor model confirmation	Preclinical evidence only; immune relevance and clinical safety remain unclear	(278)
DUB-IN-3	BHT101, 8305C and FRO cell lines;BHT101-derived xenografts in nude mice	USP8↓, GPR34↓ / ferroptosis	Genetic pathway validation with xenograft tumor model confirmation	Downstream ferroptosis mechanism unclear; no clinical validation	(263)
Fe³^+^Cur-PFP@IR780-LIP (FCIPL) nanoplatform	BHT-101 cells;BHT-101–derived xenografts in BALB/c nude mice	GPX4↓,HO-1↑	Nanoparticle-mediated ferroptosis validation with animal tumor model confirmation	Ultrasound-parameter dependence; translation of deep-tumor penetration & delivery in humans unproven.	(265)
Shikonin	CAL62 and BHT101 cells;CAL62-derived xenografts in BALB/c nude mice	GPX4 ↓ / TXNRD1 ↓;ROS ↑;ferroptosis ↑	In vitro functional validation with xenograft tumor model confirmation	Evidence limited to in vitro ATC models and nude mouse xenografts; no clinical validation.	(261)
GPX4 inhibitors (RSL-3/ML-162/ML-210)	Human PTC cell lines K1 MDA-T32 and MDA-T68	GPX4↓ / ferroptosis	In vitro pharmacologic target validation across mutation-defined models	Differential responses were observed across mutations and GPX4 inhibitors in vitro, warranting further in vivo validation.	(177)
RSL-3	Human PTC cell lines K1 and TPC-1, MDA-T32 and MDA-T68	GPX4↓, mTOR↓ / ferroptosis	In vitro pharmacologic validation with pathway-associated confirmation	Preclinical evidence is limited to in vitro models; in vivo relevance remains unclear.	(176)

GPX4, glutathione peroxidase 4; HO-1, heme oxygenase-1; xCT, cystine/glutamate antiporter (SLC7A11); TFR, transferrin receptor; PTGS2, prostaglandin-endoperoxide synthase 2; TXNRD1, thioredoxin reductase 1; ROS, reactive oxygen species; GSH, glutathione; MDA, malondialdehyde; Nrf2, nuclear factor erythroid 2–related factor 2; NQO1, NAD(P)H quinone dehydrogenase 1; PERK, protein kinase R-like endoplasmic reticulum kinase; eIF2α, eukaryotic initiation factor 2α; ATF4, activating transcription factor 4; mTOR, mechanistic target of rapamycin; USP8, ubiquitin-specific protease 8; GPR34, G protein-coupled receptor 34; ER stress, endoplasmic reticulum stress; RSL-3, Ras-selective lethal 3; ML-162/ML-210, small-molecule GPX4 inhibitors; DUB-IN-3, USP8 inhibitor; FCIPL, Fe³^+^Cur-PFP@IR780-LIP; ATC, anaplastic thyroid carcinoma; PTC, papillary thyroid carcinoma.

"↓", increase.

"↑", decrease.

Drug exposure levels and *in vivo* feasibility constitute especially prominent barriers in the translational development of ferroptosis-based strategies. Several naturally derived compounds, including curcumin (251), vitamin C (19), shikonin (261), and Tenacissoside H (262), have been reported to induce or potentiate ferroptosis-associated phenotypes in specific experimental models; however, these effects are often achieved at relatively high treatment concentrations, the systemic attainability and safety of which remain insufficiently validated *in vivo*. In parallel, small-molecule GPX4 inhibitors such as RSL-3, ML-162, and ML-210 are widely employed as mechanistic tools, yet their pharmacokinetic properties, tissue distribution, and potential toxicities in thyroid cancer–relevant animal models have not been systematically characterized, substantially limiting their prospects for clinical advancement (177).

Beyond single-agent approaches, several emerging intervention paradigms further underscore the gap between conceptual innovation and translational evidence. For instance, studies centered on deubiquitination regulatory axes have implicated the USP8–GPR34 signaling pathway in the establishment of ferroptosis tolerance, with the USP8 inhibitor DUB-IN-3 demonstrating anti-tumor activity in preclinical settings (263). Nevertheless, such findings remain restricted to a small number of experimental systems, and their applicability across different thyroid cancer subtypes, as well as their long-term therapeutic effects, requires further validation. Similarly, combination strategies integrating ferroptosis induction with conventional chemotherapy—such as the co-administration of isobavachalcone and doxorubicin—are currently supported primarily by preliminary preclinical observations, without systematic evaluation of dosing sequence, cumulative toxicity risk, or impact on tumor recurrence control (264).

In addition, nanomedicine-based strategies offer novel technical avenues for ferroptosis intervention but simultaneously introduce additional layers of translational complexity (265). Ultrasound-responsive nanoplatforms exemplified by FCIPL are designed to enhance intratumoral drug penetration and amplify ferroptotic effects within a theranostic framework. However, their therapeutic performance is highly dependent on external physical parameters, including ultrasound energy, tissue penetration depth, and spatial targeting precision, all of which may differ substantially between experimental models and human thyroid tumors. At present, studies exploring such platforms in thyroid cancer remain limited, and their clinical operability and reproducibility have yet to be rigorously assessed.

Taken together, although ferroptosis-centered strategies demonstrate measurable anti-tumor potential at the preclinical stage, their translation into clinical application is constrained by multiple challenges, including limited model representativeness, insufficient pharmacokinetic and safety data, and increased technical complexity. Future investigations should move beyond isolated mechanistic validation toward research paradigms that more closely reflect clinical reality, thereby clarifying the true therapeutic value of ferroptosis across distinct thyroid cancer subtypes.

## 8 Conclusion and prospect

Ferroptosis is an iron-dependent form of regulated cell death and has been increasingly studied in cancer biology. Thyroid cancer, as one of the most common endocrine malignancies, remains challenging in clinical management despite advances in surgery, radioactive iodine ablation, and TSH suppression, largely due to recurrence and therapeutic resistance. Accumulating evidence indicates that ferroptosis participates in the initiation and progression of thyroid cancer. In general, induction of ferroptosis in thyroid cancer cells can suppress cell proliferation and invasion, thereby restraining tumor progression.

Based on the evidence summarized in this review, ferroptosis is not only involved in the progression of thyroid cancer, but also represents a potential therapeutic target that connects molecular regulation, tumor immune microenvironment, prognostic assessment, and treatment strategies. Rather than acting as a uniform mechanism shared across different tumors, ferroptosis in thyroid cancer appears to be closely related to its biological characteristics, including redox regulation, iron metabolism, tumor differentiation status, and immune context. Taken together, these findings suggest that ferroptosis may influence both tumor progression and treatment response in a context-dependent manner, which may partly explain the heterogeneous observations reported among different thyroid cancer subtypes and experimental models.

From a mechanistic perspective, variability in ferroptosis sensitivity among thyroid cancer models can be understood as a consequence of oncogene-defined cellular states that determine susceptibility to lipid peroxidation–driven damage (266, 267). Evidence from BRAF-driven systems indicates that oncogenic signaling and its therapeutic inhibition remodel redox buffering capacity, membrane lipid composition, and iron availability in a coordinated manner, thereby positioning tumor cells at different distances from a ferroptotic tipping point. In melanoma models, pharmacologic inhibition of BRAF disrupts antioxidant homeostasis, increases mitochondrial oxidative activity and reactive oxygen species production, and is accompanied by lipid metabolic remodeling characterized by enrichment of polyunsaturated fatty acid–containing phospholipids that are intrinsically prone to peroxidation. Rather than directly triggering ferroptosis, these changes weaken upstream protective mechanisms and render cells more permissive to lipid peroxide accumulation (266, 267).

Direct experimental evidence in thyroid cancer supports this framework in the context of BRAFV600E-mutant anaplastic thyroid carcinoma (268). In these models, induction of ferroptosis through inhibition of GPX4 or blockade of system Xc^-^ leads to pronounced lipid peroxidation accompanied by elevated reactive oxygen species and intracellular iron accumulation. Combined targeting of BRAF signaling and ferroptosis pathways further amplifies lipid peroxide accumulation relative to either intervention alone, consistent with sensitization to ferroptotic cell death. Reduced expression of the iron exporter ferroportin and increased labile iron reinforce oxidative reactions, effectively lowering the threshold for ferroptosis execution. Together, these findings suggest that ferroptosis susceptibility in BRAFV600E-associated thyroid cancer reflects a shift in the balance between lipid peroxide generation and cellular protective capacity, rather than activation of a single molecular switch (266, 268).

Beyond BRAFV600E-associated settings, other recurrent genetic alterations in thyroid cancer may also influence ferroptosis susceptibility indirectly by shaping broader metabolic states and stress-adaptive programs. RAS mutations, which are frequently observed in follicular-patterned and anaplastic thyroid cancers, have been associated with metabolic remodeling in thyroid cancer, such as enhanced glucose uptake, glycolytic flux and amino acid metabolism that support tumor growth and survival, suggesting a shift in cellular energy balance and stress adaptations in oncogene-driven contexts (269, 270). RET/PTC rearrangements are common in papillary thyroid carcinoma and activate downstream proliferative and survival pathways, contributing to altered metabolic phenotypes and aggressive behavior in thyroid tumors (271). Activation of PI3K–AKT signaling, often co-occurring with other driver mutations, sustains proliferative programs in thyroid cancer and has been implicated in broader metabolic rewiring and therapeutic resistance, indicating enhanced tolerance to cellular stress (269, 272). Although direct mechanistic evidence linking these alterations to ferroptosis regulation in thyroid cancer remains limited, their established roles in metabolic reprogramming, altered lipid handling, and adaptive regulation of reactive oxygen species provide a biological rationale for considering their potential contribution to ferroptosis sensitivity, warranting further investigation in genotype-matched models (269, 270, 272).

From the mechanism level, ferroptosis and multiple core signalling pathways of thyroid cancer are intertwined. GPX4 and SLC7A11 are generally considered to be key inhibitors of ferroptosis. Their excessive expression in thyroid cancer is associated with accelerated tumour proliferation, enhanced metastasis potential and poor prognosis. On the contrary, inhibiting these molecules by genetic or drug means can restore the sensitivity of cells to ferroptosis, thus inhibiting tumour growth. NCOA4-mediated ferritin autophagy and the accompanying iron ions released further exacerbate the susceptibility of cells to ferroptosis, while regulatory non-coding RNA finely regulates ferroptosis through complex competitive endogenous RNA networks. Overall, these findings depict a precisely regulated ferroptosis pattern in thyroid cancer, suggesting that targeted treatment for these nodes may block the progression of the tumour. However, the dual and even sometimes contradictory role of regulatory factors such as p53 highlights the situational dependence of ferroptosis, which needs to be explored more deeply.

The interplay between ferroptosis and the TME constitutes an emerging and complex field that needs to be further studied. On the one hand, ferroptosis in tumour cells can promote the death of immunogenic cells and enhance anti-tumour immunity, which is mainly achieved by releasing lipid peroxide and damage-related molecular patterns. For instance, CD8+ T cells mediate ferroptosis in thyroid cancer cells by releasing IFNγ, thus enhancing their inherent cytotoxicity. Conversely, heightened ferroptosis within effector immune subsets, including CD8+ T cells or NK cells, can undermine immune surveillance; while the same process in regulatory T cells (Tregs) amplifies anti-tumor immunity. Similarly, macrophage polarisation and the activity of medullary inhibitory cells are also deeply affected by ferroptosis signals. These results highlight the two-way effects of ferroptosis in the TME, indicating that the treatment strategy must be carefully weighed: not only to induce ferroptosis in tumour cells, but also to protect beneficial immune function. Importantly, the bidirectional effects of ferroptosis in the TME are further complicated by pronounced cellular heterogeneity, encompassing malignant cells, immune populations, and stromal components with distinct metabolic and redox states. Conventional bulk transcriptomic or metabolic analyses are inherently limited in their ability to resolve such heterogeneity. In this context, single-cell RNA sequencing (scRNA-seq) has emerged as a powerful approach to dissect ferroptosis-relevant heterogeneity at cell-type resolution. Recent scRNA-seq studies in thyroid cancer have delineated diverse tumor and immune cell populations and uncovered substantial intratumoral and microenvironmental diversity in metabolic programs, oxidative stress responses, and immune regulatory pathways. By enabling high-resolution mapping of cell-specific transcriptional states, scRNA-seq provides an opportunity to identify ferroptosis-prone tumor subpopulations, characterize immune cell states with differential sensitivity to lipid peroxidation and oxidative stress, and clarify how metabolic crosstalk within the TME shapes context-dependent ferroptotic vulnerability. Together, these insights may refine our understanding of ferroptosis regulation beyond bulk tumor analyses and offer a conceptual framework for more precise modulation of ferroptosis in the tumor microenvironment (273, 274).

The clinical transformation of ferroptosis in thyroid cancer is still in its infancy, but the initial progress is encouraging. Ferroptosis-related genes (such as GPX4, AKR1C3 and DPP4) have shown their potential as prognostic biomarkers. Their prediction models can achieve risk stratification and be associated with immune infiltration patterns. Such models, especially those that integrate ferroptosis-related long-chain non-coding RNA, provide new insights for individualised prognostic evaluation and treatment guidance. However, most models are still at the computational level, and strong external verification and clinical trial evidence support are still needed before they can be widely used. The spatial heterogeneity of ferroptosis-related gene expression and the functional verification at the protein level are still important gaps in current research. In this context, patient-derived organoid models are increasingly recognized as a promising translational platform to bridge computational predictions with functional validation. Organoids established from primary thyroid tumors preserve key genetic, phenotypic, and metabolic characteristics of the original lesions, thereby providing a physiologically relevant system for ex vivo assessment of ferroptosis-related vulnerabilities (275). By enabling direct evaluation of ferroptosis-inducing agents or combination strategies under controlled conditions, organoid models offer an opportunity to functionally test patient-specific therapeutic sensitivities that cannot be captured by transcriptomic analyses alone. Moreover, recent studies have demonstrated the feasibility of using thyroid cancer organoids to inform individualized treatment decisions through drug sensitivity profiling (276). Collectively, these approaches may help address current gaps related to spatial heterogeneity and protein-level functional validation, and facilitate the translation of ferroptosis-based strategies toward more personalized clinical applications.

In terms of treatment, regulating ferroptosis provides an opportunity to overcome drug resistance and incurable diseases. Whether it is natural compounds such as curumin, vitamin C, neferine, or synthetic derivatives or approved drugs such as Anlotinib, they have been proven to induce ferroptosis in the thyroid cancer model. These compounds elicit their effects through multiple mechanisms, including promoting the accrual of reactive oxygen species and ferritinophagy, alongside the simultaneous suppression of cellular antioxidant defenses. This underscores the therapeutic heterogeneity of ferroptosis induction strategies. Importantly, the joint strategy is particularly promising. Ferroptosis inducers demonstrate synergy with immune checkpoint inhibitors, radiotherapy, and targeted therapies, thereby potentiating anti-tumor efficacy. Concurrently, the deployment of nanocarrier systems offers a means to enhance drug accumulation at the tumor site while mitigating systemic toxicity. Despite these advances, the conversion to clinical treatment regimens is still hampered by factors such as limited in ivo research, uncertain optimal dose, and potential systemic toxicity.

Several limitations must be recognised. At present, the understanding of ferroptosis in thyroid cancer mainly comes from *in vitro* models, small-animal studies or bioinformatics analyses, and there is relatively little clinical evidence. The heterogeneity between thyroid cancer subtypes, especially between indolent papillary thyroid carcinoma and aggressive anaplastic or poorly differentiated thyroid cancer, has not been fully solved in studies focusing on ferroptosis. In addition, before designing safe and effective treatment interventions, it is necessary to carefully consider the context-dependent roles of ferroptosis in regulating immunity and tumour progression.

Future research on ferroptosis in thyroid cancer still requires experimental systems that more closely reflect clinical heterogeneity. Patient-derived thyroid cancer organoids or primary cultures could provide a practical platform to evaluate ferroptosis induction under defined genetic backgrounds, such as BRAF-mutant or RAS-mutant tumors. These models would allow direct testing of ferroptosis inducers, either alone or in combination with commonly used targeted agents, and facilitate assessment of treatment sensitivity, resistance patterns, and dose-dependent effects.

In parallel, further *in vivo* studies using genetically characterized mouse models or patient-derived xenografts are needed to clarify how ferroptosis modulation influences tumor progression and immune responses within an intact tumor microenvironment. Given the differential sensitivity of tumor cells and immune cell subsets to ferroptotic stress, future work should carefully examine treatment timing and cell-type specificity. At the clinical level, ferroptosis-related molecular signatures should be evaluated in prospective cohorts and interpreted alongside established clinicopathological risk stratification systems, rather than as standalone predictors. Such efforts may help determine whether ferroptosis-based biomarkers can contribute to treatment selection, response monitoring, or combination therapy strategies in thyroid cancer.
